# The global burden of chronic kidney disease due to diabetes mellitus type 2 attributable to diet high in sugar-sweetened beverages among the elderly: a comprehensive analysis from 1990 to 2021

**DOI:** 10.3389/fnut.2025.1615351

**Published:** 2025-06-19

**Authors:** Ben Hu, Xiaohan Qiu, Zhengbiao Luo, Jingxiong Chen, Jun Feng, Linlin Hou

**Affiliations:** ^1^Department of Cardiology, The Second People's Hospital of Hefei, The Affiliated Hefei Hospital of Anhui Medical University, Hefei, China; ^2^The Fifth Clinical Medical School of Anhui Medical University, Hefei, China; ^3^Department of Cardiology, Shanghai Ninth People's Hospital, Shanghai Jiaotong University School of Medicine, Shanghai, China; ^4^Department of Gastroenterology, The First Affiliated Hospital of USTC, Division of Life Sciences and Medicine, University of Science and Technology of China, Hefei, China

**Keywords:** sugar-sweetened beverages, chronic kidney disease, type 2 diabetes mellitus, elderly, global burden of disease

## Abstract

**Background:**

The consumption of sugar-sweetened beverages (SSBs) has been linked to numerous health complications, including chronic kidney disease due to type 2 diabetes mellitus (CKD-T2DM). However, the global burden of CKD-T2DM attributable to high SSB consumption among elderly populations remains poorly characterized.

**Methods:**

Using data from the Global Burden of Disease Study 2021, we examined age-standardized mortality rates (ASMR) and disability-adjusted life year rates (ASDR) of CKD-T2DM attributable to high SSB consumption among individuals aged 60 years and older across 204 countries and territories from 1990 to 2021. We employed joinpoint regression analysis to assess temporal trends and conducted decomposition analysis to quantify the contributions of population growth, aging, and epidemiological changes to the observed burden.

**Results:**

Globally, the ASMR of CKD-T2DM attributable to high SSB consumption among elderly increased from 0.21 (95% UI: 0.10–0.38) per 100,000 in 1990 to 0.37 (95% UI: 0.18–0.62) per 100,000 in 2021, with an average annual percent change (AAPC) of 1.89% (95% CI: 1.47–2.31). The ASDR similarly increased from 4.5 (95% UI: 2.12–7.66) to 7.24 (95% UI: 3.49–11.69) per 100,000. We observed pronounced socioeconomic and geographical disparities, with high socio-demographic index (SDI) regions experiencing the highest burden and fastest increase. Notably, decomposition analysis revealed that population growth was the primary driver of increased burden globally, while epidemiological changes played a more dominant role in high SDI regions.

**Conclusion:**

Our findings highlight a substantial and increasing burden of CKD-T2DM attributable to high SSB consumption among elderly populations globally, with distinct patterns across socioeconomic development levels. These results underscore the importance of targeted interventions to reduce SSB consumption, particularly in regions experiencing rapid increases in disease burden, as part of comprehensive strategies to address the growing challenge of diet-related kidney disease in aging populations.

## Introduction

In an era when health systems worldwide are grappling with the dual challenge of aging populations and growing chronic disease burden, dietary factors have emerged as critical yet modifiable determinants of public health (1, 2). Recent global health initiatives, including the World Health Organization’s 2023–2030 acceleration plan to reduce noncommunicable diseases (3), have specifically highlighted the urgent need to address unhealthy diets as a primary risk factor for chronic conditions. Among these dietary concerns, sugar-sweetened beverage (SSB) consumption stands out as a particularly troubling trend with far-reaching health implications (4, 5).

The consumption of SSBs has increased dramatically over recent decades, transcending geographical and socioeconomic boundaries. This global shift in dietary patterns coincides with rising rates of obesity, type 2 diabetes mellitus (T2DM), and their associated complications (6, 7). Of particular concern is chronic kidney disease due to diabetes mellitus type 2 (CKD-T2DM), a devastating complication that significantly impacts quality of life, healthcare costs, and mortality, especially among elderly populations (8–10). The intersection of population aging, changing dietary patterns, and chronic disease represents one of the most pressing public health challenges of our time.

While previous research has extensively documented the association between SSB consumption and T2DM, the specific burden of CKD-T2DM attributable to high SSB intake among elderly populations has received limited attention in global health literature. This knowledge gap is particularly concerning given that elderly individuals are more vulnerable to both the development and progression of kidney disease due to age-related physiological changes and cumulative exposure to risk factors (11–13). Furthermore, as the global population of adults aged 60 years and older is projected to double by 2050 (14), understanding and addressing modifiable risk factors for CKD-T2DM in this demographic has become increasingly crucial for sustainable healthcare planning.

Recent policy initiatives in several countries, including sugar taxes on beverages in Mexico, the United Kingdom, and parts of the United States, reflect growing recognition of SSBs as a public health concern (4, 15). However, the effectiveness of such interventions requires robust evidence regarding the specific disease burdens attributable to SSB consumption across different populations and regions. This evidence is particularly vital in the context of competing health priorities and limited resources that characterize many healthcare systems today.

While the association between high SSB consumption and CKD-T2DM is well-documented in general populations, critical gaps persist in understanding its burden among elderly individuals—a demographic projected to double by 2050. Existing studies lack granularity in assessing how socioeconomic development modulates this burden across decomposition of demographic and epidemiological drivers. Furthermore, temporal trends and attributable drivers remain unexplored at a global scale. Our study aims to address this critical knowledge gap by providing a multilevel global, regional, and national analysis of the burden of CKD-T2DM attributable to high SSB consumption among elderly populations from 1990 to 2021. By leveraging data from the Global Burden of Disease (GBD) 2021 study, we examine the temporal trends, geographical patterns, and sociodemographic determinants of this burden, offering insights that can inform targeted public health interventions and policy formulation. These contributions provide actionable insights for aging societies navigating the dual challenges of nutritional transition and chronic disease escalation.

## Methods

### Data sources

GBD 2021 project, supported by over 11,500 collaborators from 164 countries, provides a comprehensive assessment of global health status and disease burden. This monumental effort involves extensive data collection, review, and analysis. Detailed information on data sources can be accessed through the GBD 2021 Data Input Sources tool on the Institute for Health Metrics and Evaluation website (https://ghdx.healthdata.org/gbd-2021/sources) (16). We retrieved estimates of death, and disability-adjusted life years rates rate for chronic kidney disease due to diabetes mellitus type 2 attributable to diet high in sugar-sweetened beverages, along with their 95% uncertainty intervals (UI), for 8 age groups (60–64, 65–69, 70–74, 75–79, 80–84, 85–89, 90–94, 95+ years) from 1990 to 2021 using the Global Health Data Exchange (GHDx) query tool (https://vizhub.healthdata.org/gbd-results/) (17). The uncertainty intervals were calculated per GBD methodology by extracting the 25th and 975th percentiles from 1,000 statistically independent model simulations. Regarding GBD data processing and modeling process are available in Supplementary Methods.

### Patient and public involvement

For the use of identified data in the GBD study, the University of Washington Institutional Review Board granted a waiver of informed consent, as this research did not involve individual participants. Ethical approval details are available at: https://www.healthdata.org/research-analysis/gbd (18).

### Definition of socio-demographic index (SDI)

The SDI is a metric that evaluates a country or region’s level of development based on fertility rates, educational attainment, and per capita income, on a scale from 0 to 1. Higher SDI values denote greater socioeconomic development. Research has shown that SDI correlates with variations in disease prevalence and mortality rates, highlighting the interplay between socioeconomic factors and health outcomes. Countries and territories were categorized into five SDI quintiles (low, low-middle, middle, middle-high, and high) to examine the association between chronic kidney disease due to diabetes mellitus type 2 attributable to diet high in sugar-sweetened beverages among elderly and socioeconomic development (17, 18).

### Definition of disability-adjusted life years (DALYs)

DALYs represent a composite metric combining mortality-related years lost (YLLs) and morbidity-associated years lived with disability (YLDs), quantifying population health gaps (Supplementary Methods) (19).

### Definition of CKD-T2DM

CKD-T2DM is defined according to the International Classification of Diseases, 9th edition (ICD-9): 250.40, 250.42 and 10th edition (ICD-10): E11.2, E11.21, E11.22, E11.29 (18). Complete technical documentation of source datasets and corresponding metadata descriptors can be retrieved from the online data platform at: http://ghdx.healthdata.org/gbd-2021/data-input-sources (18).

### Definition of diet high in sugar-sweetened beverages

A diet high in sugar-sweetened beverages was defined as the consumption of beverages containing 226.8 kcal or more per 50-g serving (measured in grams per day). This category includes carbonated drinks, sodas, energy drinks, and fruit-flavored beverages but excludes 100% pure fruit and vegetable juices. The GBD database integrates data from various national and international dietary surveys, food consumption databases, and nutritional studies. The modeling process inherently accounts for cultural variations and regional consumption patterns, ensuring that the classification of dietary risk factors accurately reflects distinct dietary behaviors across different countries and regions (17).

### Statistical analysis

First, to estimate age-standardized mortality rates (ASMR), and disability-adjusted life years rates (ASDR) of chronic kidney disease due to diabetes mellitus type 2 attributable to diet high in sugar-sweetened beverages among elderly, direct age standardization was employed. Second, joinpoint regression analysis was used to assess trends in ASMR, and ASDR (Supplementary Methods). Third, in addition, to derive an in-depth understanding of explanatory factors driving changes in chronic kidney disease due to diabetes mellitus type 2 attributable to diet high in sugar-sweetened beverages among elderly (death, and DALYs) from 1990 to 2021, we conducted decomposition analyses by population size, age structure, and epidemiologic changes (Supplementary Methods). Statistical analyses were performed using R versions 4.3.0 (http://www.r-project.org). Statistical significance was set at 2-sided *p* < 0.05 (17).

## Results

### Global and SDI-specific trends in CKD due to DM2 attributable to diet high in sugar-sweetened beverages among elderly

Our analysis revealed substantial increases in both mortality and disease burden attributable to SSB consumption among elderly populations between 1990 and 2021. Globally, the ASMR attributable to diet high in SSB increased from 0.21 (95% UI: 0.10 to 0.38) per 100,000 in 1990 to 0.37 (95% UI: 0.18 to 0.62) per 100,000 in 2021, representing an average annual percent change (AAPC) of 1.89% (95% CI: 1.47 to 2.31). Similarly, the ASDR rate increased from 4.5 (95% UI: 2.12 to 7.66) per 100,000 in 1990 to 7.24 (95% UI: 3.49 to 11.69) per 100,000 in 2021, with an AAPC of 1.61% (95% CI: 1.26 to 1.97) (Table 1).

**Table 1:** ASMR and ASDR of chronic kidney disease due to diabetes mellitus type 2 attributable to diet high in sugar-sweetened beverages among elderly in 1990 and 2021, and change from 1990 to 2021 at the global and regional level.

Location	ASMR, per 100,000 (95% UI)		AAPC (95% CI)	ASDR, per 100,000 (95% UI)		AAPC (95% CI)
	1990	2021		1990	2021	
Global	0.21 (0.1 to 0.38)	0.37 (0.18 to 0.62)	1.89 (1.47 to 2.31)	4.5 (2.12 to 7.66)	7.24 (3.49 to 11.69)	1.61 (1.26 to 1.97)
High SDI region	0.29 (0.12 to 0.54)	0.58 (0.26 to 1.02)	2.41 (1.96 to 2.86)	6.75 (2.97 to 12.1)	11.76 (5.39 to 20.26)	1.92 (1.65 to 2.2)
High-middle SDI region	0.19 (0.08 to 0.34)	0.24 (0.11 to 0.43)	0.84 (0.68 to 1)	3.8 (1.77 to 6.61)	4.73 (2.17 to 8.2)	0.69 (0.61 to 0.77)
Middle SDI region	0.19 (0.09 to 0.34)	0.37 (0.17 to 0.65)	2.35 (2.03 to 2.67)	3.7 (1.75 to 6.44)	7.38 (3.39 to 12.56)	2.39 (2.18 to 2.6)
Low-middle SDI region	0.11 (0.05 to 0.19)	0.19 (0.08 to 0.33)	1.94 (1.8 to 2.08)	2.3 (1.05 to 4.06)	3.95 (1.8 to 6.7)	1.84 (1.74 to 1.95)
Low SDI region	0.11 (0.05 to 0.21)	0.12 (0.05 to 0.22)	0.39 (0.23 to 0.55)	2.35 (1.01 to 4.25)	2.4 (1.06 to 4.31)	0.17 (0.07 to 0.27)
Andean Latin America	0.73 (0.29 to 1.39)	1.19 (0.46 to 2.35)	1.72 (1.44 to 1.99)	13.99 (5.56 to 26.43)	23.6 (9.17 to 45.27)	1.74 (1.53 to 1.96)
Australasia	0.09 (0.04 to 0.19)	0.13 (0.05 to 0.29)	1.25 (0.78 to 1.73)	3.36 (1.36 to 6.66)	3.75 (1.52 to 7.23)	0.4 (0.1 to 0.7)
Caribbean	0.41 (0.18 to 0.79)	0.67 (0.28 to 1.24)	1.65 (1.3 to 2)	8.08 (3.42 to 15.29)	12.8 (5.48 to 23.59)	1.57 (1.21 to 1.92)
Central Asia	0.04 (0.01 to 0.09)	0.09 (0.03 to 0.17)	2.4 (0.67 to 4.16)	2.27 (0.86 to 4.6)	2.91 (1.27 to 5.21)	0.65 (0.25 to 1.06)
Central Europe	0.13 (0.05 to 0.25)	0.12 (0.05 to 0.23)	−0.2 (−0.79 to 0.39)	3.65 (1.63 to 6.34)	3.56 (1.66 to 6.06)	0.02 (−0.25 to 0.3)
Central Latin America	0.64 (0.24 to 1.34)	0.93 (0.34 to 1.88)	1.63 (1.06 to 2.2)	12.69 (4.92 to 25.76)	19.51 (7.33 to 38.08)	1.72 (1.14 to 2.29)
Central Sub-Saharan Africa	0.34 (0.11 to 0.83)	0.21 (0.07 to 0.49)	−1.56 (−1.67 to −1.45)	7.01 (2.38 to 16.72)	4.08 (1.46 to 9.37)	−1.73 (−1.83 to −1.63)
East Asia	0.08 (0.03 to 0.16)	0.19 (0.07 to 0.4)	2.88 (2.67 to 3.08)	1.39 (0.53 to 2.8)	3.71 (1.4 to 7.63)	3.2 (2.83 to 3.58)
Eastern Europe	0.02 (0.01 to 0.04)	0.05 (0.02 to 0.12)	3.58 (2.68 to 4.5)	1.07 (0.42 to 2.11)	1.59 (0.64 to 3.11)	1.14 (0.81 to 1.47)
Eastern Sub-Saharan Africa	0.18 (0.07 to 0.36)	0.26 (0.1 to 0.51)	1.16 (1.08 to 1.23)	3.47 (1.37 to 6.89)	4.67 (1.86 to 8.99)	0.99 (0.92 to 1.06)
High-income Asia Pacific	0.42 (0.14 to 0.9)	0.36 (0.13 to 0.79)	−0.38 (−0.79 to 0.02)	7.35 (2.61 to 15.14)	6.62 (2.57 to 13.6)	−0.45 (−0.79 to −0.11)
High-income North America	0.39 (0.14 to 0.83)	1.19 (0.49 to 2.25)	3.83 (3.35 to 4.31)	9.66 (3.65 to 19.45)	23.74 (9.85 to 43.79)	3.01 (2.57 to 3.45)
North Africa and Middle East	0.33 (0.13 to 0.66)	0.41 (0.17 to 0.8)	0.79 (0.41 to 1.17)	6.24 (2.56 to 12.11)	7.92 (3.42 to 14.74)	0.73 (0.33 to 1.13)
Oceania	0.29 (0.12 to 0.55)	0.55 (0.23 to 1.09)	2.08 (1.8 to 2.35)	5.6 (2.32 to 10.66)	10.22 (4.2 to 20.48)	1.98 (1.79 to 2.17)
South Asia	0.08 (0.03 to 0.14)	0.15 (0.06 to 0.27)	2.13 (1.83 to 2.43)	1.84 (0.82 to 3.3)	3.36 (1.43 to 6.01)	1.9 (1.42 to 2.38)
Southeast Asia	0.11 (0.05 to 0.22)	0.3 (0.13 to 0.56)	3.24 (3.05 to 3.43)	2.28 (0.98 to 4.29)	5.96 (2.55 to 10.69)	3.17 (2.96 to 3.38)
Southern Latin America	1.06 (0.4 to 2.15)	0.94 (0.35 to 1.93)	−0.13 (−0.51 to 0.25)	20.23 (7.92 to 39.53)	17.4 (6.93 to 34.16)	−0.24 (−0.58 to 0.09)
Southern Sub-Saharan Africa	0.15 (0.05 to 0.35)	0.23 (0.08 to 0.5)	1.37 (0.49 to 2.27)	3.89 (1.53 to 7.85)	5.23 (2.12 to 10.24)	0.96 (0.45 to 1.47)
Tropical Latin America	0.45 (0.17 to 0.94)	0.81 (0.31 to 1.59)	2.01 (1.54 to 2.47)	9.14 (3.56 to 18.31)	16.08 (6.5 to 30.36)	1.81 (1.35 to 2.28)
Western Europe	0.21 (0.09 to 0.4)	0.24 (0.11 to 0.44)	0.59 (0.27 to 0.9)	5.12 (2.32 to 8.99)	5 (2.38 to 8.73)	−0.01 (−0.14 to 0.12)
Western Sub-Saharan Africa	0.06 (0.02 to 0.11)	0.11 (0.04 to 0.23)	2.23 (2.15 to 2.31)	1.23 (0.53 to 2.37)	2.34 (0.95 to 4.57)	2.16 (2.09 to 2.24)

ASMR, age-standardized death rate; ASDR, age-standardized disability-adjusted life years rates; SDI, sociodemographic index; UI, uncertainty interval; AAPC, average annual percent change; CI, confidence interval.

**Figure 1:**
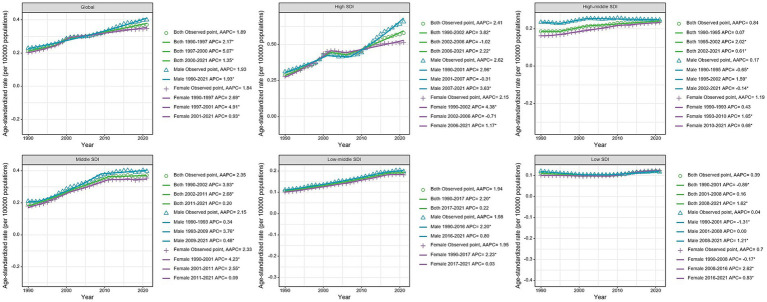
Joinpoint regression analysis of age-standardized death rates of chronic kidney disease due to diabetes mellitus type 2 attributable to diet high in sugar-sweetened beverages among elderly at the Global and 5 SDI regions from 1990 to 2021. *p*-value *p < 0.05.

**Figure 2:**
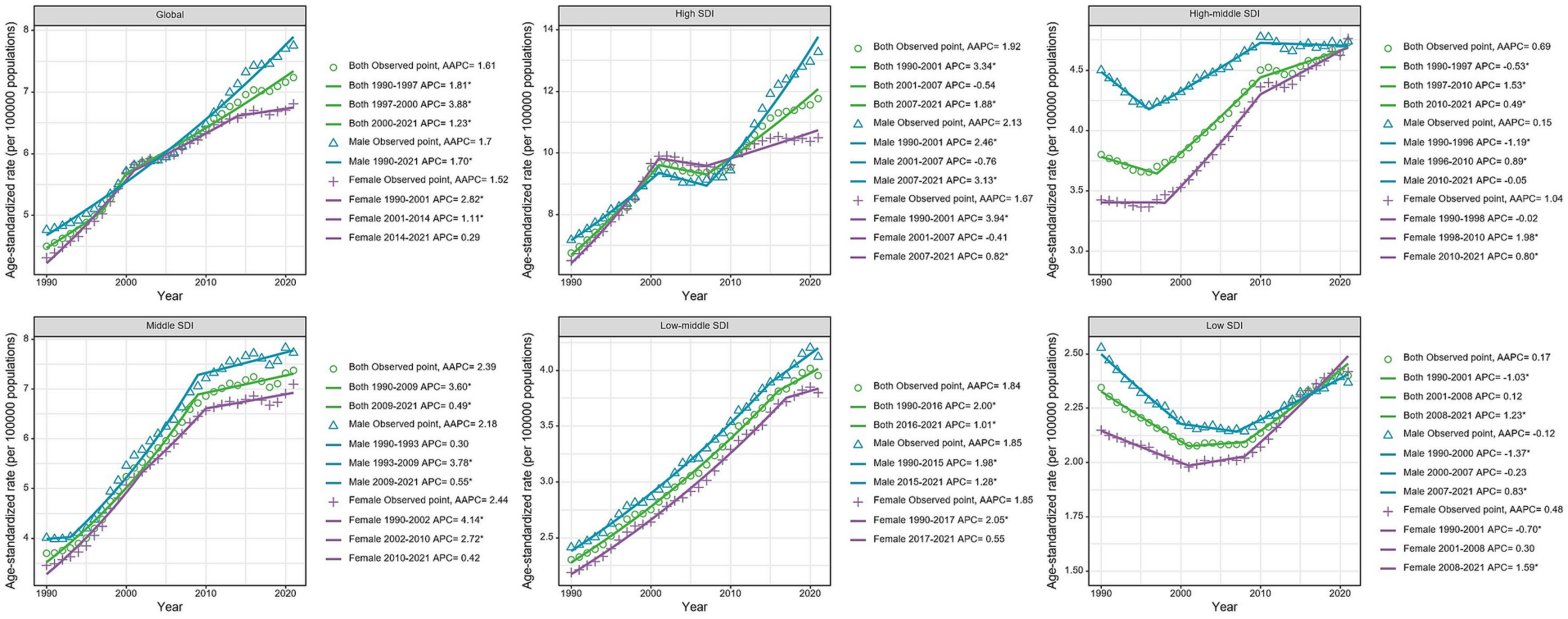
Joinpoint regression analysis of age-standardized disability-adjusted life years rates of chronic kidney disease due to diabetes mellitus type 2 attributable to diet high in sugar-sweetened beverages among elderly at the Global and 5 SDI regions from 1990 to 2021. *p*-value *p < 0.05.

**Figure 3:**
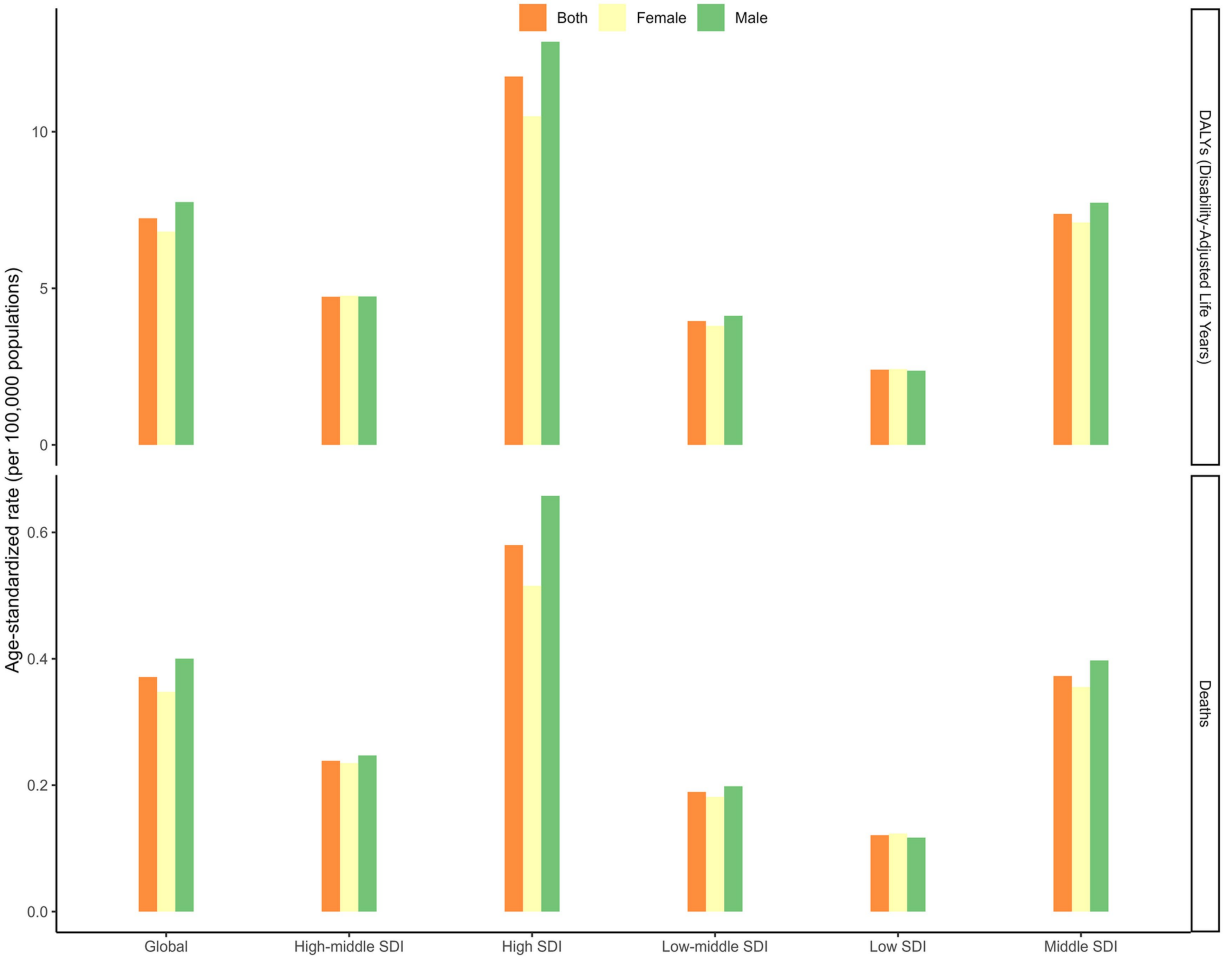
Age-standardized death, disability-adjusted life years rate of chronic kidney disease due to diabetes mellitus type 2 attributable to diet high in sugar-sweetened beverages among elderly in SDI regions in 2021.

**Figure 4:**
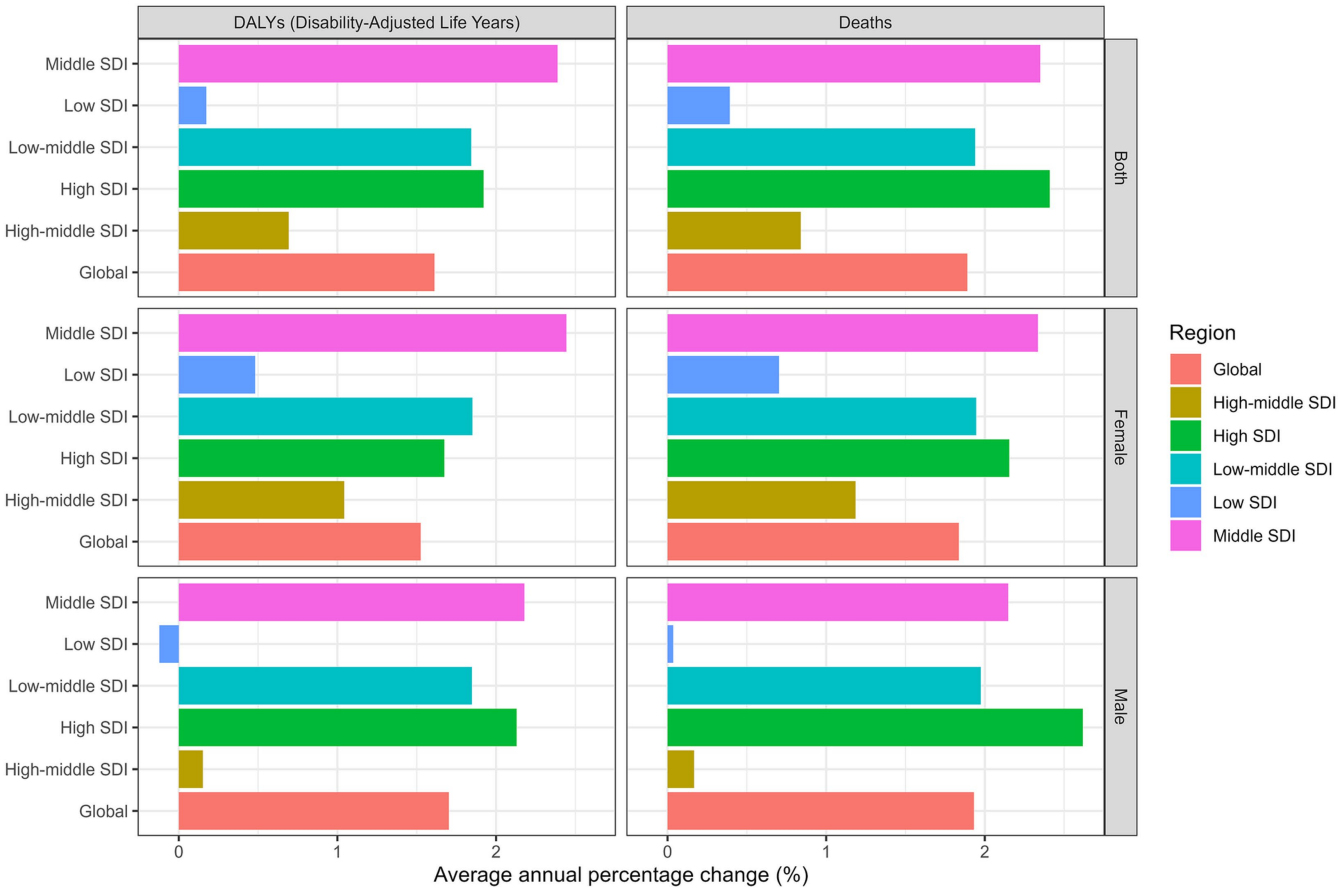
Average annual percent change of age-standardized death, and disability-adjusted life years rate from 1990 to 2021 in SDI regions.

The joinpoint regression analysis revealed significant upward trends in both ASMR and ASDR globally and across most SDI regions (Figures 1, 2). The trends showed distinct patterns with several inflection points, indicating periods of acceleration or deceleration in the rate of increase over the study period. These inflection points were particularly evident around 1997–2000, 2007–2010, and 2014–2017 across different SDI regions. For instance, in the high SDI category, three clear segments were identified (1990–2002, 2002–2006, and 2006–2021) with annual percentage change (APC) of 3.82, −1.02, and 2.22 respectively, demonstrating initial rapid growth followed by a brief deceleration and subsequent reacceleration. When analyzed by SDI regions, the high SDI regions experienced the highest burden in 2021, with an ASMR of 0.58 (95% UI: 0.26 to 1.02) per 100,000 and an ASDR of 11.76 (95% UI: 5.39 to 20.26) per 100,000 (Table 1 and Figure 3). These regions also demonstrated the most substantial increase in mortality with an AAPC of 2.41% (95% CI: 1.96 to 2.86; Figure 4). In contrast, low SDI regions showed the smallest increase in both mortality and disease burden, with an AAPC of only 0.39% (95% CI: 0.23 to 0.55) for ASMR and 0.17% (95% CI: 0.07 to 0.27) for ASDR (Table 1). As shown in Supplementary Figures 1 and 2, there was a clear positive association between SDI level and both ASMR and ASDR across 204 countries, indicating higher disease burden in more developed countries. In additional, our analysis of age-standardized rates by sex revealed consistent patterns across SDI regions, with males generally experiencing higher rates of both mortality and DALYs compared to females, particularly in more developed regions (Figure 3).

### Regional distribution and trends

**Figure 5:**
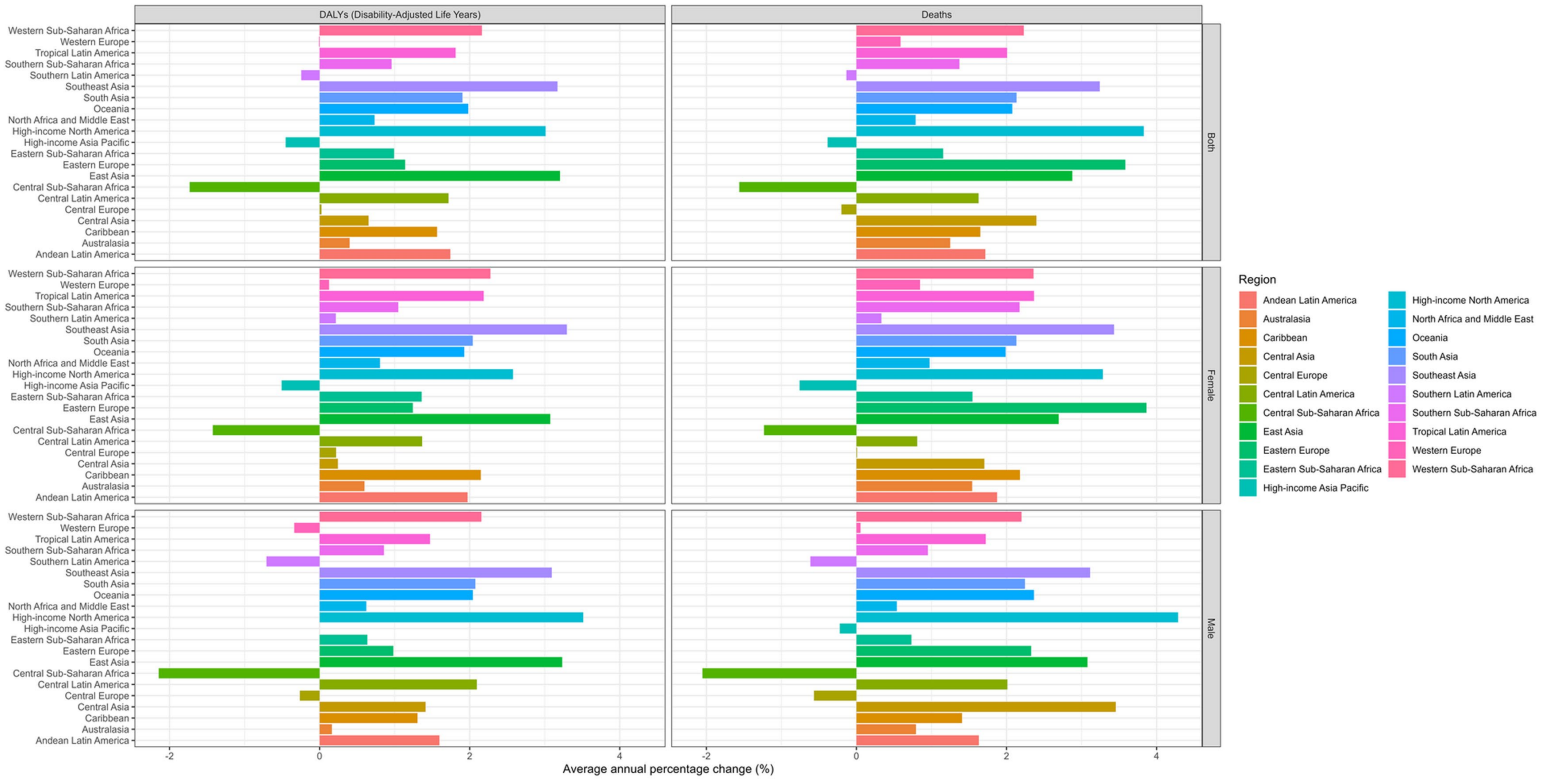
Average annual percent change of age-standardized death, and disability-adjusted life years rate from 1990 to 2021 in 21 regions.

Among the 21 GBD regions, Southern Latin America had the highest ASMR in 1990 at 1.06 (95% UI: 0.40 to 2.15) per 100,000, followed by Andean Latin America at 0.73 (95% UI: 0.29 to 1.39) per 100,000. By 2021, High-income North America surpassed all regions with an ASMR of 1.19 (95% UI: 0.49 to 2.25) per 100,000, demonstrating the steepest increase in mortality with an AAPC of 3.83% (95% CI: 3.35 to 4.31) (Table 1). Figure 5 illustrates the AAPC in ASMR and ASDR across all 21 regions, showing the wide range of regional changes during the study period.

Notably, several regions experienced decreases in both mortality and disease burden. Central Sub-Saharan Africa showed the most significant reduction in ASMR with an AAPC of −1.56% (95% CI: −1.67 to −1.45) and in ASDR with an AAPC of −1.73% (95% CI: −1.83 to −1.63). Other regions with declining trends included High-income Asia Pacific (AAPC for ASMR: −0.38%; 95% CI: −0.79 to 0.02), Central Europe (AAPC for ASMR: −0.20%; 95% CI: −0.79 to 0.39), and Southern Latin America (AAPC for ASMR: −0.13%; 95% CI: −0.51 to 0.25) (Table 1 and Figure 5). The most substantial increases in disease burden were observed in Southeast Asia and East Asia, with AAPCs for ASDR of 3.17% (95% CI: 2.96 to 3.38) and 3.20% (95% CI: 2.83 to 3.58), respectively. Eastern Europe exhibited the highest increase in mortality with an AAPC for ASMR of 3.58% (95% CI: 2.68 to 4.50), although starting from a low baseline of 0.02 (95% UI: 0.01 to 0.04) per 100,000 in 1990 (Table 1). Supplementary Figure 3 demonstrates a moderate positive correlation (*R* = 0.30, *p* < 0.001) between SDI and disease burden metrics, with High-income North America and Latin American regions showing notably higher values at similar SDI levels compared to regions like Eastern Europe and Central Asia. Additionally, Supplementary Figure 4 reveals similar regional patterns with a slightly stronger correlation (*R* = 0.33, *p* < 0.001), highlighting the considerable heterogeneity in disease burden across regions with similar socio-demographic development, suggesting that factors beyond socioeconomic status significantly influence regional disease patterns.

### Geographic distribution and country-level burden

**Figure 6:**
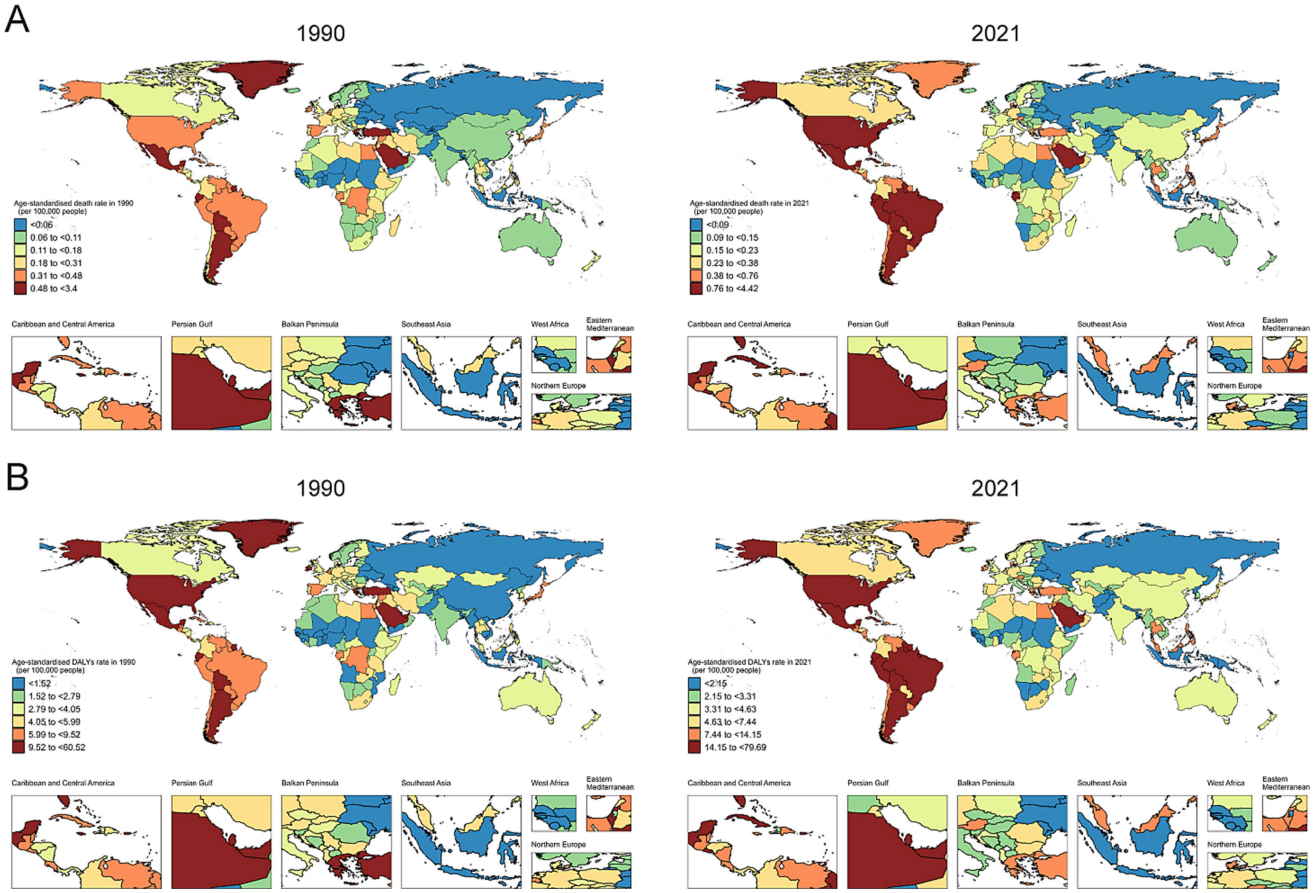
Maps showing **(A)** age-standardized death rates, **(B)** age-standardized disability-adjusted life years rate of chronic kidney disease due to diabetes mellitus type 2 attributable to diet high in sugar-sweetened beverages among elderly, in 204 countries and territories, between 1990 and 2021.

**Figure 7:**
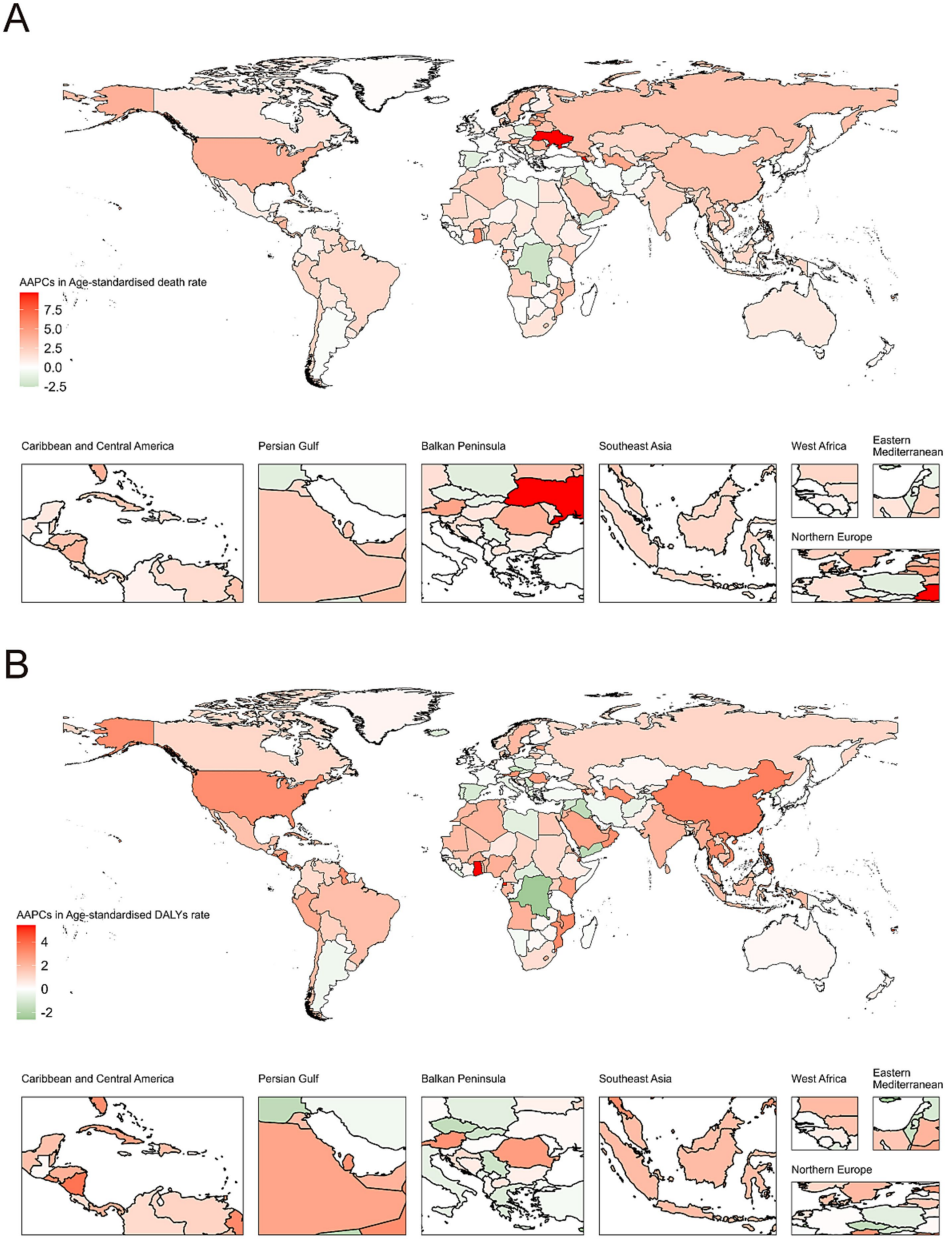
World map of AAPCs in **(A)** age-standardized death rates, **(B)** age-standardized disability-adjusted life years rate of chronic kidney disease due to diabetes mellitus type 2 attributable to diet high in sugar-sweetened beverages among elderly from 1990 to 2021. AAPC, average annual percentage change.

The geographical distribution of CKD due to DM2 attributable to SSB consumption revealed substantial heterogeneity. Figures 6A, B illustrate the global distribution of ASMR and ASDR in 2021, respectively, showing notably higher burdens in North America (ASMR of 1.19 per 100,000), Andean Latin America (1.19), and Southern Latin America (0.94), with moderate burdens in Central Latin America (0.93) and the Caribbean (0.67). The world maps of AAPC (Figures 7A, B) further highlight regions with the most rapid increases in burden, with High-income North America (AAPC of 3.83%), Eastern Europe (3.58%), and Southeast Asia (3.24%) showing the most concerning upward trends. Notably, Central Sub-Saharan Africa experienced the most significant decrease (AAPC −1.56%) while East Asia showed a substantial increase (2.88%) despite its relatively lower absolute burden. The age-standardized rates demonstrated a clear association with economic development, with higher rates typically observed in more developed countries where SSB consumption has been historically more prevalent. However, our temporal analysis revealed a concerning pattern: while high SDI countries maintained the highest absolute burden, the rate of increase was accelerating more rapidly in many middle and low-middle SDI countries, suggesting a potential future shift in the global distribution of burden.

At the country level, the burden varied substantially, as detailed in Supplementary Table 1. In 2021, the highest ASMRs were observed in American Samoa (4.42 per 100,000), Northern Mariana Islands (3.21 per 100,000), and Mauritius (2.51 per 100,000). Conversely, countries like Tajikistan, Ukraine, and Belarus had the lowest ASMRs (all below 0.01 per 100,000). The most dramatic increases in ASMR between 1990 and 2021 were seen in Armenia (AAPC: 9.45%), Ukraine (AAPC: 9.72%), and Ghana (AAPC: 5.42%).

### Decomposition analysis of disease burden changes

**Figure 8:**
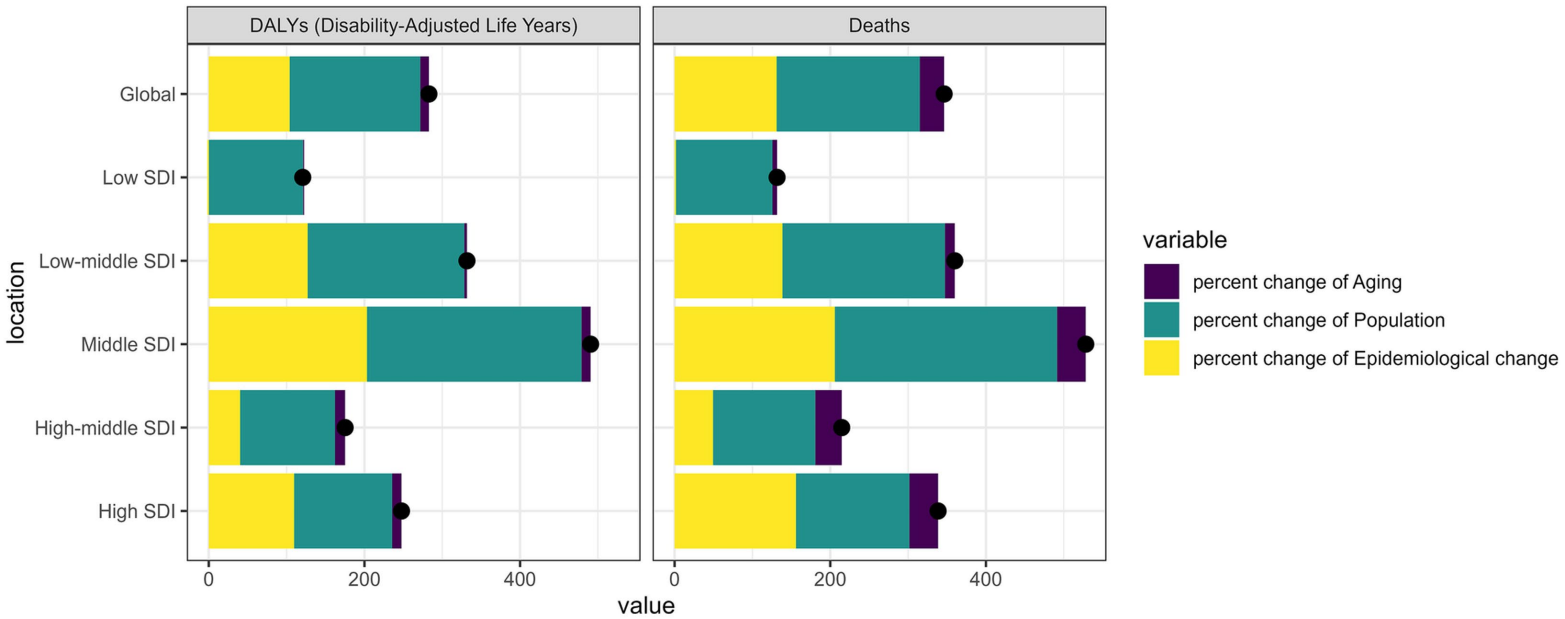
Changes in deaths, and disability-adjusted life years percentage change of chronic kidney disease due to diabetes mellitus type 2 attributable to diet high in sugar-sweetened beverages among elderly. According to population-level determinants of population growth, aging, and epidemiological change from 1990 to 2021 globally and 5 SDI regions.

Decomposition analysis of the changes in disease burden (Supplementary Table 2 and Figure 8) revealed the relative contributions of different factors to the overall increase. Globally, population growth accounted for the largest proportion of the increase in both deaths (183.84%) and DALYs (167.72%), followed by epidemiological changes (131.11% for deaths and 103.89% for DALYs), while population aging contributed relatively less (31.36% for deaths and 11.23% for DALYs).

Interestingly, the pattern varied significantly across SDI regions (Figure 8). In high SDI regions, epidemiological changes played a more dominant role, contributing 155.9% to the increase in deaths, compared to low SDI regions where epidemiological factors contributed only 1.74%. Conversely, in low SDI regions, population growth was the primary driver, accounting for 123.93% of the increase in deaths. These findings highlight the different underlying mechanisms driving the burden increase across development levels. According to Supplementary Table 2, the contribution of aging to disease burden varied substantially across different SDI regions. In high SDI regions, aging accounted for a larger proportion of the increase in mortality, reflecting the more advanced stage of demographic transition in these countries. In contrast, in low SDI regions, aging contributed less to the mortality increase, with population growth being the predominant factor.

## Discussion

Our comprehensive analysis reveals a concerning global trend: CKD-T2DM attributable to high sugar-sweetened beverage consumption among elderly populations has increased substantially from 1990 to 2021. This burden disproportionately affects high SDI regions but is accelerating in middle and low-middle SDI countries, pointing to an emerging public health crisis that demands immediate attention.

The COVID-19 pandemic exposed critical vulnerabilities in healthcare systems worldwide, particularly in managing chronic diseases among elderly populations (20, 21). As countries rebuild and strengthen their health infrastructures in 2025, our findings provide timely evidence for integrating dietary interventions into post-pandemic health policies. The recent World Health Organization’s 2023–2030 acceleration plan to reduce noncommunicable diseases specifically emphasizes reducing unhealthy diet consumption (3), aligning perfectly with our findings on SSB-related disease burden. The substantial economic burden of CKD-T2DM on healthcare systems comes at a particularly challenging time, as many countries face fiscal constraints following pandemic-related expenditures. The Global Economic Forum’s 2025 projections indicate that healthcare costs associated with diet-related chronic diseases will reach unprecedented levels, potentially compromising economic recovery efforts in both high and middle-income nations (22).

Our findings revealing the highest disease burden in high SDI regions contradict traditional patterns of chronic disease distribution but align with contemporary understanding of nutritional transition (23–25). The rapid increase in burden observed in middle SDI regions, however, signals an alarming trend that could exacerbate existing health inequities as these countries simultaneously manage communicable disease burdens and healthcare system limitations (26). The variable contribution of population aging, growth, and epidemiological factors across different SDI regions underscores the need for tailored intervention strategies. Low SDI regions, where population growth primarily drives burden increases, require different approaches than high SDI regions where epidemiological changes dominate. This nuanced understanding is crucial given the recent UN Sustainable Development Goals mid-point assessment, which highlighted concerning gaps in progress toward health equity targets.

Our analysis shows that in all SDI regions, the ASMR and ASDR of CKD-T2DM attributable to high SSB consumption in elderly men are consistently higher than those in women. This difference may be jointly caused by biological, behavioral, and sociocultural factors. Estrogen can reduce oxidative stress and inflammation, thereby delaying the progression of CKD in women, while androgens in men may exacerbate insulin resistance and glomerular hyperfiltration (27). In addition, men have higher visceral fat content, which increases the susceptibility to metabolic syndrome and diabetic nephropathy even at similar body mass index levels (28). Global dietary surveys show that men consume 20–30% more SSB than women, especially energy drinks and soda, while elderly women are more likely to seek routine care for diabetes management, thus detecting and alleviating the development of chronic kidney disease earlier (29, 30).

Our decomposition analysis showed that epidemiological changes drive burden increases in high SDI regions, suggesting that even wealthy nations cannot sustain the growing healthcare costs without preventive interventions (31, 32). These results provide compelling evidence supporting regulatory measures to reduce SSB consumption (33–35). The effectiveness of sugar taxes implemented in Mexico, the United Kingdom, and parts of the United States is now corroborated by our findings showing particularly high disease burdens in regions without such interventions (36). The WHO’s 2024 guidelines on fiscal policies for health improvement specifically cite SSB taxation as a high-impact, cost-effective intervention, which our study now quantitatively supports with elderly-specific outcomes data.

Recent cohort studies reveals a robust dose–response relationship between SSB consumption and the burden of CKD-T2DM among elderly populations, such as Heo et al., which demonstrated that daily SSB intake exceeding 350 mL significantly elevates CKD risk in elderly individuals (9). Experimental models further indicate that chronic SSB consumption exacerbates oxidative stress and inflammation in renal tissues, accelerating CKD progression in diabetic populations (37). This underscore the importance of defining harmful consumption thresholds for public health guidance. The escalating burden of CKD-T2DM attributable to SSBs demands urgent, targeted policy interventions. Evidence from jurisdictions implementing SSB taxes provides compelling support for fiscal measures. For instance, Mexico’s 2014 tax of 1 peso per liter reduced SSB purchases by 12% nationally, with the most significant declines observed in low-income households (38). Over a decade, this policy is projected to avert 189,000 CKD cases, highlighting its cost-effectiveness and scalability. Similarly, South Africa’s Health Promotion Levy decreased SSB consumption by 29% among elderly populations, demonstrating that fiscal policies can effectively target high-risk demographics (39).

The global food and beverage industry’s recent commitments to reduce sugar content in products, while promising, appear insufficient given the accelerating disease burden we observed. The International Food & Beverage Alliance’s 2023 pledge to reduce sugar by 15% by 2030 falls short of the reduction levels our data suggest would be necessary to meaningfully impact disease outcomes (40). As governments worldwide consider more stringent regulatory frameworks in 2025, our findings provide critical evidence to counter industry resistance.

The digital health revolution accelerated by the pandemic provides novel opportunities to address SSB consumption (41, 42). The European Union’s “Digital Health for Aging Populations” initiative and similar programs in the United States and Japan could integrate SSB reduction components based on our evidence of its substantial impact on kidney health (43). Artificial intelligence applications in personalized nutrition, which have seen remarkable advancement in 2024, offer promising approaches to modify dietary behaviors based on individual risk profiles (44, 45). Our region-specific findings could inform the development of these technologies to prioritize areas with the highest or most rapidly increasing disease burden.

While our study provides robust global estimates, several limitations warrant mention. First, in many low SDI regions, particularly in Sub-Saharan Africa, access to routine screening and advanced diagnostic tools remains limited that our estimates represent the reported burden, which may not capture the true epidemiological reality (46). The GBD dataset may underestimate SSB consumption in regions where traditional, locally produced sugar-sweetened drinks are common but not captured in standardized surveys. In high SDI regions, longer life expectancy and improved management of diabetes in these regions may inflate CKD-T2DM burden estimates by increasing the proportion of elderly individuals living with advanced chronic conditions (47). Second, our analysis does not account for potential confounding factors such as concurrent changes in healthcare access, diagnostic capabilities, and overall dietary patterns that might influence CKD-T2DM rates independently of SSB consumption. Future research should explore the dose–response relationship between SSB consumption and CKD-T2DM in elderly populations, as well as the potential interaction effects with other dietary factors, physical activity, and genetic predisposition. Additionally, prospective studies examining the impact of SSB reduction interventions specifically on elderly kidney health outcomes would complement our retrospective analysis.

## Conclusion

As population aging accelerates worldwide, addressing modifiable risk factors for chronic diseases becomes increasingly urgent. Our study demonstrates that reducing SSB consumption represents a concrete, evidence-based target for intervention that could substantially impact the global burden of kidney disease among elderly populations. In an era of competing health priorities and limited resources, such targeted approaches based on robust evidence are essential for effective and sustainable public health action.

## Data Availability

The datasets presented in this study can be found in online repositories. The names of the repository/repositories and accession number(s) can be found in the article/Supplementary material.
