# Supplementary material for: The global burden of chronic kidney disease due to diabetes mellitus type 2 attributable to diet high in sugar-sweetened beverages among the elderly: a comprehensive analysis from 1990 to 2021
Source: Front Nutr. 2025 Jun 19;12:1615351. doi: 10.3389/fnut.2025.1615351 (PMC12221943; doi:10.3389/fnut.2025.1615351)
Supplement: Supplementary file 1 [file Table_1.DOCX]

**Supplementary Material**

Table of Contents

**Supplementary Table 1.** Age-standardized deaths, and DALYs rate of chronic kidney disease due to diabetes mellitus type 2 attributable to diet high in sugar-sweetened beverages among elderly in 1990 and 2021, and change from 1990 to 2021 at 204 countries level.

**Supplementary Table 2.** Changes in deaths, and DALYs rate of chronic kidney disease due to diabetes mellitus type 2 attributable to diet high in sugar-sweetened beverages among elderly according to population-level determinants from 1990 to 2021 globally and 5 SDI regions.

**Supplementary Fig. 1** Association between age-standardized deaths rate of chronic kidney disease due to diabetes mellitus type 2 attributable to diet high in sugar-sweetened beverages among elderly and 204 countries.

**Supplementary Fig. 2** Association between age-standardized DALYs rate of chronic kidney disease due to diabetes mellitus type 2 attributable to diet high in sugar-sweetened beverages among elderly and 204 countries.

**Supplementary Fig. 3** Association between age-standardized deaths rate of chronic kidney disease due to diabetes mellitus type 2 attributable to diet high in sugar-sweetened beverages among elderly and 21 regions.

**Supplementary Fig. 4** Association between age-standardized DALYs rate of chronic kidney disease due to diabetes mellitus type 2 attributable to diet high in sugar-sweetened beverages among elderly and 21 regions.

**This supplemental material has been provided by the authors to give readers additional information about their work.**

**Supplementary Table 1.** Age-standardized deaths, and DALYs rate of chronic kidney disease due to diabetes mellitus type 2 attributable to diet high in sugar-sweetened beverages among elderly in 1990 and 2021, and change from 1990 to 2021 at 204 countries level.

| location | ASMR, per 100,000 (95% UI) | | AAPC (95% CI) | ASDR, per 100,000 (95% UI) | | AAPC (95% CI) |
| --- | --- | --- | --- | --- | --- | --- |
|  | 1990 | 2021 |  | 1990 | 2021 |  |
| Afghanistan | 0.1 (0.03 to 0.28) | 0.08 (0.02 to 0.24) | -0.57 (-0.81 to -0.32) | 2.21 (0.6 to 5.63) | 1.76 (0.43 to 4.84) | -0.58 (-0.81 to -0.35) |
| Albania | 0.16 (0.04 to 0.39) | 0.15 (0.04 to 0.37) | 0.43 (-0.47 to 1.33) | 4.09 (1.29 to 9.58) | 4.06 (1.38 to 8.9) | 0.32 (-0.38 to 1.03) |
| Algeria | 0.15 (0.04 to 0.44) | 0.32 (0.08 to 0.87) | 2.47 (2.31 to 2.63) | 2.77 (0.73 to 7.92) | 5.44 (1.39 to 14.43) | 2.12 (1.95 to 2.29) |
| American Samoa | 1.31 (0.34 to 3.38) | 4.42 (1.14 to 11.42) | 4.2 (3.9 to 4.51) | 24.26 (6.31 to 62.47) | 79.69 (21.12 to 203.46) | 4.09 (3.82 to 4.36) |
| Andorra | 0.61 (0.17 to 1.48) | 0.3 (0.08 to 0.75) | -2.3 (-2.65 to -1.96) | 12.59 (3.97 to 28.29) | 6.73 (2 to 15.08) | -2.03 (-2.31 to -1.75) |
| Angola | 0.07 (0.02 to 0.17) | 0.14 (0.04 to 0.38) | 2.39 (2.27 to 2.51) | 1.49 (0.45 to 3.68) | 2.89 (0.8 to 7.54) | 2.18 (2.07 to 2.29) |
| Antigua and Barbuda | 0.22 (0.07 to 0.56) | 0.37 (0.12 to 0.88) | 1.54 (0.79 to 2.31) | 4.23 (1.35 to 10.69) | 6.68 (2.18 to 15.81) | 1.24 (0.42 to 2.07) |
| Argentina | 1.37 (0.49 to 2.84) | 1.16 (0.39 to 2.53) | -0.25 (-0.65 to 0.14) | 25.84 (9.48 to 51.66) | 21.51 (7.83 to 44.64) | -0.37 (-0.72 to -0.01) |
| Armenia | 0 (0 to 0) | 0.04 (0.01 to 0.09) | 9.45 (8.15 to 10.77) | 0.43 (0.14 to 1.02) | 1.18 (0.41 to 2.8) | 3.71 (3.06 to 4.37) |
| Australia | 0.09 (0.03 to 0.19) | 0.13 (0.04 to 0.29) | 1.14 (0.55 to 1.74) | 3.36 (1.26 to 6.98) | 3.71 (1.4 to 7.39) | 0.19 (-0.15 to 0.54) |
| Austria | 0.19 (0.06 to 0.43) | 0.66 (0.23 to 1.4) | 4.55 (3.39 to 5.72) | 4.71 (1.6 to 10.32) | 10.97 (4.09 to 22.05) | 3.01 (2.32 to 3.7) |
| Azerbaijan | 0.03 (0.01 to 0.08) | 0.07 (0.02 to 0.18) | 3.09 (2.72 to 3.47) | 1.41 (0.4 to 3.87) | 2.64 (0.75 to 6.31) | 2.04 (1.79 to 2.29) |
| Bahamas | 0.33 (0.09 to 0.86) | 0.36 (0.11 to 0.93) | 0.35 (-0.6 to 1.3) | 6.79 (1.92 to 17.23) | 6.77 (2.09 to 17.34) | -0.03 (-0.74 to 0.69) |
| Bahrain | 0.42 (0.11 to 1.1) | 0.87 (0.21 to 2.38) | 2.43 (1.97 to 2.9) | 7.79 (2.14 to 19.73) | 15.29 (3.79 to 41.1) | 2.26 (1.95 to 2.56) |
| Bangladesh | 0.02 (0.01 to 0.05) | 0.03 (0.01 to 0.07) | 1.2 (0.6 to 1.8) | 0.43 (0.13 to 1.07) | 0.61 (0.18 to 1.48) | 1.07 (0.75 to 1.39) |
| Barbados | 0.73 (0.2 to 1.85) | 0.75 (0.21 to 1.94) | 0.17 (-0.86 to 1.2) | 14.29 (3.86 to 35.29) | 13.17 (3.83 to 34.1) | -0.19 (-1.24 to 0.88) |
| Belarus | 0 (0 to 0.01) | 0.01 (0 to 0.03) | 3.12 (0.19 to 6.13) | 0.76 (0.19 to 2.12) | 0.87 (0.24 to 2.28) | 0.38 (0.13 to 0.64) |
| Belgium | 0.31 (0.1 to 0.69) | 0.29 (0.1 to 0.65) | 0.28 (-0.62 to 1.19) | 7.71 (2.72 to 15.89) | 7.1 (2.68 to 14.44) | 0.04 (-0.52 to 0.59) |
| Belize | 0.38 (0.11 to 0.99) | 0.75 (0.22 to 1.78) | 2.24 (1.53 to 2.96) | 7.6 (2.19 to 19.59) | 14.06 (4.11 to 34.05) | 2.05 (1.34 to 2.76) |
| Benin | 0.05 (0.01 to 0.12) | 0.09 (0.03 to 0.22) | 2.04 (1.87 to 2.2) | 0.94 (0.28 to 2.37) | 1.73 (0.53 to 4.32) | 2.01 (1.82 to 2.2) |
| Bermuda | 0.48 (0.14 to 1.15) | 0.27 (0.08 to 0.7) | -1.77 (-2.35 to -1.19) | 9.07 (2.62 to 21.82) | 4.9 (1.43 to 12.51) | -2.05 (-2.42 to -1.68) |
| Bhutan | 0.04 (0.01 to 0.11) | 0.07 (0.02 to 0.18) | 1.51 (1.36 to 1.65) | 0.96 (0.29 to 2.34) | 1.4 (0.4 to 3.67) | 1.27 (1.13 to 1.42) |
| Bolivia (Plurinational State of) | 0.94 (0.25 to 2.41) | 1.44 (0.4 to 3.48) | 1.42 (1.29 to 1.56) | 17.85 (4.77 to 44.85) | 26.66 (7.27 to 64.2) | 1.33 (1.22 to 1.44) |
| Bosnia and Herzegovina | 0.11 (0.03 to 0.28) | 0.14 (0.04 to 0.34) | 0.89 (0.19 to 1.58) | 2.73 (0.71 to 6.92) | 3.26 (1.03 to 7.48) | 0.55 (-0.13 to 1.24) |
| Botswana | 0.07 (0.02 to 0.19) | 0.09 (0.02 to 0.24) | 0.53 (0.09 to 0.97) | 1.86 (0.54 to 4.77) | 2.07 (0.63 to 5.33) | 0.2 (-0.11 to 0.52) |
| Brazil | 0.45 (0.16 to 0.95) | 0.82 (0.31 to 1.61) | 2.02 (1.55 to 2.49) | 9.19 (3.54 to 18.56) | 16.28 (6.55 to 30.8) | 1.83 (1.36 to 2.3) |
| Brunei Darussalam | 0.36 (0.09 to 1.03) | 0.38 (0.1 to 1.01) | 0.08 (-0.2 to 0.36) | 5.96 (1.61 to 16.37) | 6.49 (1.8 to 17.33) | 0.17 (-0.01 to 0.36) |
| Bulgaria | 0.16 (0.06 to 0.35) | 0.21 (0.07 to 0.47) | 1.04 (0.22 to 1.86) | 5.12 (1.95 to 10.24) | 5.32 (1.86 to 11.61) | 0.44 (-0.03 to 0.91) |
| Burkina Faso | 0.04 (0.01 to 0.1) | 0.08 (0.02 to 0.21) | 2.3 (2.12 to 2.48) | 0.83 (0.24 to 2.02) | 1.63 (0.47 to 4.15) | 2.27 (2.12 to 2.42) |
| Burundi | 0.17 (0.05 to 0.47) | 0.16 (0.04 to 0.44) | -0.26 (-0.36 to -0.15) | 3.23 (0.85 to 8.75) | 2.85 (0.76 to 7.76) | -0.4 (-0.5 to -0.3) |
| Cabo Verde | 0.02 (0.01 to 0.05) | 0.05 (0.02 to 0.13) | 3.28 (2.51 to 4.06) | 0.48 (0.15 to 1.12) | 1.03 (0.32 to 2.51) | 2.57 (1.82 to 3.33) |
| Cambodia | 0.06 (0.02 to 0.15) | 0.1 (0.03 to 0.27) | 1.78 (1.66 to 1.89) | 1.28 (0.4 to 3.14) | 2.16 (0.62 to 5.55) | 1.7 (1.59 to 1.82) |
| Cameroon | 0.12 (0.04 to 0.3) | 0.19 (0.05 to 0.51) | 1.16 (0.83 to 1.48) | 2.4 (0.72 to 5.67) | 3.75 (1.01 to 9.99) | 1.29 (1.01 to 1.56) |
| Canada | 0.16 (0.04 to 0.4) | 0.24 (0.07 to 0.57) | 1.36 (0.93 to 1.79) | 3.34 (0.96 to 8.18) | 4.68 (1.42 to 10.65) | 1.14 (0.8 to 1.48) |
| Central African Republic | 0.17 (0.05 to 0.45) | 0.14 (0.04 to 0.37) | -0.68 (-0.87 to -0.49) | 3.68 (1.1 to 9.44) | 2.87 (0.78 to 7.34) | -0.79 (-1.02 to -0.56) |
| Chad | 0.03 (0.01 to 0.08) | 0.04 (0.01 to 0.11) | 1.24 (1.15 to 1.33) | 0.65 (0.21 to 1.59) | 0.92 (0.27 to 2.34) | 1.22 (1.13 to 1.3) |
| Chile | 0.3 (0.09 to 0.7) | 0.54 (0.19 to 1.13) | 2.3 (1.61 to 3) | 6.04 (1.88 to 13.76) | 9.78 (3.56 to 19.49) | 1.88 (1.24 to 2.53) |
| China | 0.07 (0.02 to 0.14) | 0.18 (0.06 to 0.4) | 3.24 (2.95 to 3.54) | 1.21 (0.44 to 2.55) | 3.58 (1.31 to 7.56) | 3.49 (3.23 to 3.74) |
| Colombia | 0.23 (0.07 to 0.51) | 0.29 (0.1 to 0.63) | 0.68 (0.23 to 1.14) | 4.94 (1.72 to 10.41) | 6.75 (2.4 to 13.7) | 1.06 (0.5 to 1.61) |
| Comoros | 0.24 (0.06 to 0.65) | 0.27 (0.07 to 0.73) | 0.22 (0.09 to 0.36) | 4.56 (1.12 to 11.96) | 4.8 (1.24 to 12.89) | 0.12 (-0.02 to 0.25) |
| Congo | 0.22 (0.06 to 0.58) | 0.21 (0.06 to 0.57) | -0.05 (-0.18 to 0.08) | 4.44 (1.2 to 11.35) | 4.05 (1.15 to 10.42) | -0.29 (-0.56 to -0.03) |
| Cook Islands | 0.53 (0.14 to 1.43) | 1.63 (0.44 to 3.91) | 3.74 (3.48 to 4) | 10.15 (2.74 to 27.27) | 30.08 (8.22 to 70.88) | 3.63 (3.39 to 3.87) |
| Costa Rica | 0.39 (0.11 to 0.96) | 0.61 (0.18 to 1.41) | 1.44 (0.54 to 2.35) | 9.5 (2.63 to 22.03) | 12.94 (3.94 to 29.54) | 1.03 (0.26 to 1.81) |
| Coted'Ivoire | 0.09 (0.03 to 0.24) | 0.1 (0.03 to 0.26) | 0.37 (0.24 to 0.5) | 1.98 (0.59 to 5.05) | 2.19 (0.64 to 5.43) | 0.38 (0.23 to 0.53) |
| Croatia | 0.12 (0.04 to 0.3) | 0.18 (0.05 to 0.41) | 1.93 (0.15 to 3.74) | 3.53 (1.09 to 7.95) | 4.06 (1.38 to 8.74) | 1.07 (-0.31 to 2.46) |
| Cuba | 0.36 (0.1 to 0.85) | 0.81 (0.24 to 1.85) | 2.88 (2.36 to 3.4) | 7.62 (2.15 to 18.23) | 15.82 (4.69 to 35.95) | 2.59 (2.08 to 3.11) |
| Cyprus | 0.52 (0.14 to 1.27) | 0.23 (0.06 to 0.61) | -2.47 (-2.85 to -2.1) | 8.08 (2.37 to 18.81) | 3.91 (1.09 to 9.53) | -2.3 (-2.53 to -2.08) |
| Czechia | 0.11 (0.03 to 0.28) | 0.07 (0.02 to 0.18) | -0.99 (-1.81 to -0.17) | 3.59 (1.03 to 8.21) | 2.17 (0.67 to 4.77) | -1.6 (-2.12 to -1.07) |
| Democratic People's Republic of Korea | 0.08 (0.02 to 0.2) | 0.06 (0.02 to 0.15) | -1.01 (-1.12 to -0.9) | 1.65 (0.49 to 4.07) | 1.29 (0.38 to 3.26) | -0.79 (-0.93 to -0.64) |
| Democratic Republic of the Congo | 0.43 (0.12 to 1.1) | 0.2 (0.05 to 0.56) | -2.4 (-2.55 to -2.24) | 8.75 (2.58 to 22.19) | 4.01 (1.11 to 10.69) | -2.5 (-2.65 to -2.35) |
| Denmark | 0.15 (0.04 to 0.36) | 0.4 (0.13 to 0.86) | 3.34 (2.82 to 3.86) | 4.59 (1.4 to 10.5) | 7.79 (2.7 to 16.42) | 1.76 (1.58 to 1.93) |
| Djibouti | 0.09 (0.02 to 0.22) | 0.27 (0.07 to 0.69) | 3.71 (3.59 to 3.84) | 1.6 (0.43 to 4.08) | 4.68 (1.26 to 12.16) | 3.56 (3.44 to 3.69) |
| Dominica | 0.68 (0.21 to 1.67) | 1.13 (0.32 to 2.76) | 1.6 (1.52 to 1.68) | 12.8 (3.81 to 31.31) | 20.41 (5.75 to 51.02) | 1.5 (1.39 to 1.6) |
| Dominican Republic | 0.31 (0.09 to 0.78) | 0.47 (0.12 to 1.23) | 1.72 (1.29 to 2.15) | 5.77 (1.58 to 14.66) | 9.18 (2.41 to 23.66) | 1.82 (1.4 to 2.24) |
| Ecuador | 1.26 (0.37 to 2.85) | 1.82 (0.48 to 4.63) | 1.22 (0.75 to 1.69) | 24.98 (7.4 to 56.27) | 36.92 (9.97 to 91.89) | 1.25 (0.77 to 1.73) |
| Egypt | 0.36 (0.11 to 0.93) | 0.53 (0.18 to 1.18) | 1.4 (1.03 to 1.76) | 6.31 (2.06 to 15.87) | 10.06 (3.52 to 21.8) | 1.56 (1.21 to 1.92) |
| El Salvador | 0.35 (0.09 to 0.89) | 0.94 (0.23 to 2.35) | 3.46 (2.87 to 4.05) | 7.6 (2.11 to 19.06) | 18.89 (4.49 to 46.26) | 3.2 (2.74 to 3.67) |
| Equatorial Guinea | 0.17 (0.05 to 0.44) | 0.85 (0.17 to 2.43) | 5.53 (5.09 to 5.98) | 3.56 (1.03 to 9.06) | 15.98 (3.39 to 44.84) | 5.12 (4.69 to 5.56) |
| Eritrea | 0.11 (0.03 to 0.3) | 0.2 (0.05 to 0.56) | 1.92 (1.72 to 2.12) | 2.2 (0.58 to 5.86) | 3.61 (0.91 to 10.15) | 1.73 (1.53 to 1.93) |
| Estonia | 0.04 (0.01 to 0.1) | 0.19 (0.05 to 0.49) | 5.66 (3.87 to 7.47) | 1.94 (0.57 to 4.83) | 4.59 (1.34 to 11.05) | 2.81 (2.24 to 3.39) |
| Eswatini | 0.2 (0.05 to 0.54) | 0.2 (0.05 to 0.53) | 0.07 (-0.23 to 0.38) | 4.66 (1.2 to 11.97) | 4.27 (1.12 to 11.28) | -0.3 (-0.6 to 0) |
| Ethiopia | 0.19 (0.06 to 0.47) | 0.25 (0.07 to 0.62) | 0.79 (0.68 to 0.9) | 3.51 (1.03 to 8.49) | 4.22 (1.28 to 10.69) | 0.57 (0.45 to 0.69) |
| Fiji | 0.53 (0.13 to 1.53) | 2.23 (0.56 to 5.94) | 4.73 (4.24 to 5.23) | 10.53 (2.69 to 29.65) | 40.19 (10.2 to 107.45) | 4.42 (4.04 to 4.8) |
| Finland | 0.1 (0.03 to 0.22) | 0.12 (0.04 to 0.28) | 0.91 (0.6 to 1.21) | 2.95 (1 to 6.38) | 2.91 (0.97 to 6.61) | 0.15 (-0.18 to 0.49) |
| France | 0.24 (0.07 to 0.55) | 0.22 (0.07 to 0.47) | -0.21 (-0.64 to 0.22) | 4.69 (1.58 to 10.11) | 4.6 (1.69 to 9.53) | -0.12 (-0.6 to 0.36) |
| Gabon | 0.38 (0.1 to 1.08) | 0.79 (0.17 to 2.21) | 2.39 (2.19 to 2.58) | 7.64 (2.1 to 20.59) | 14.1 (3.19 to 38.41) | 2.02 (1.83 to 2.2) |
| Gambia | 0.08 (0.02 to 0.19) | 0.1 (0.03 to 0.25) | 0.77 (0.4 to 1.14) | 1.54 (0.47 to 3.71) | 1.93 (0.55 to 4.93) | 0.76 (0.36 to 1.16) |
| Georgia | 0.01 (0 to 0.04) | 0.07 (0.02 to 0.18) | 4.29 (2.9 to 5.7) | 1.43 (0.39 to 3.91) | 2.36 (0.67 to 5.91) | 1.48 (0.95 to 2.02) |
| Germany | 0.21 (0.07 to 0.45) | 0.29 (0.1 to 0.66) | 1.63 (0.78 to 2.49) | 5.57 (1.97 to 11.57) | 5.44 (2.03 to 11.13) | 0.08 (-0.37 to 0.53) |
| Ghana | 0.05 (0.01 to 0.13) | 0.26 (0.07 to 0.69) | 5.42 (5.27 to 5.58) | 0.96 (0.27 to 2.4) | 4.78 (1.27 to 12.4) | 5.4 (5.27 to 5.53) |
| Greece | 0.84 (0.26 to 1.87) | 0.64 (0.23 to 1.39) | -0.24 (-2.28 to 1.85) | 14.01 (4.74 to 29.61) | 11.49 (4.3 to 23.98) | -0.91 (-2.91 to 1.13) |
| Greenland | 0.5 (0.13 to 1.26) | 0.51 (0.15 to 1.17) | 0.16 (-0.05 to 0.37) | 10.05 (2.78 to 24.44) | 10.67 (3.28 to 23.62) | 0.3 (0.14 to 0.46) |
| Grenada | 0.32 (0.1 to 0.8) | 0.57 (0.18 to 1.47) | 2.37 (1.55 to 3.21) | 6.37 (1.98 to 15.78) | 10.51 (3.32 to 26.81) | 2.05 (1.45 to 2.65) |
| Guam | 1.51 (0.41 to 3.61) | 1.1 (0.29 to 2.82) | -0.69 (-1.89 to 0.52) | 29.78 (8.03 to 69.2) | 25.34 (6.82 to 64.28) | -0.18 (-1.03 to 0.68) |
| Guatemala | 0.45 (0.12 to 1.2) | 0.49 (0.14 to 1.21) | 0.01 (-0.61 to 0.64) | 8.56 (2.36 to 22.59) | 9.92 (2.94 to 24.01) | 0.22 (-0.41 to 0.85) |
| Guinea | 0.05 (0.02 to 0.13) | 0.06 (0.02 to 0.16) | 0.57 (0.25 to 0.89) | 1.18 (0.37 to 2.83) | 1.37 (0.4 to 3.51) | 0.52 (0.36 to 0.68) |
| Guinea-Bissau | 0.07 (0.02 to 0.18) | 0.06 (0.02 to 0.15) | -0.36 (-0.44 to -0.28) | 1.41 (0.4 to 3.67) | 1.23 (0.38 to 2.99) | -0.43 (-0.5 to -0.37) |
| Guyana | 0.42 (0.13 to 1.02) | 1.04 (0.31 to 2.48) | 3.06 (2.13 to 4.01) | 8.17 (2.47 to 20.06) | 20.35 (5.96 to 48.81) | 3.21 (2.29 to 4.15) |
| Haiti | 0.17 (0.05 to 0.47) | 0.16 (0.04 to 0.46) | -0.17 (-0.24 to -0.11) | 3.39 (1.01 to 9.12) | 3.12 (0.83 to 8.82) | -0.21 (-0.27 to -0.15) |
| Honduras | 0.12 (0.03 to 0.33) | 0.29 (0.07 to 0.73) | 2.99 (2.52 to 3.47) | 3.26 (0.87 to 8.53) | 6.44 (1.74 to 15.95) | 2.22 (1.58 to 2.87) |
| Hungary | 0.08 (0.03 to 0.18) | 0.11 (0.03 to 0.26) | 1.32 (0.81 to 1.84) | 2.84 (1.02 to 5.95) | 2.75 (0.97 to 5.96) | 0.08 (-0.28 to 0.44) |
| Iceland | 0.09 (0.03 to 0.22) | 0.09 (0.02 to 0.2) | -0.12 (-0.5 to 0.26) | 3.82 (1.2 to 8.55) | 2.72 (0.79 to 6.57) | -0.93 (-1.24 to -0.61) |
| India | 0.09 (0.04 to 0.16) | 0.17 (0.07 to 0.31) | 2.22 (1.9 to 2.54) | 2.11 (0.92 to 3.82) | 3.9 (1.66 to 7.05) | 2.1 (1.88 to 2.32) |
| Indonesia | 0.04 (0.01 to 0.08) | 0.07 (0.03 to 0.16) | 2.07 (1.92 to 2.22) | 0.84 (0.31 to 1.78) | 1.48 (0.56 to 3.07) | 1.89 (1.74 to 2.04) |
| Iran (Islamic Republic of) | 0.21 (0.06 to 0.55) | 0.19 (0.05 to 0.46) | -0.18 (-0.45 to 0.1) | 4.22 (1.17 to 10.97) | 3.72 (1.11 to 8.95) | -0.33 (-0.55 to -0.11) |
| Iraq | 0.26 (0.07 to 0.72) | 0.17 (0.05 to 0.45) | -1.37 (-1.88 to -0.86) | 5.47 (1.47 to 14.78) | 3.27 (0.92 to 8.47) | -1.67 (-2.1 to -1.25) |
| Ireland | 0.35 (0.11 to 0.75) | 0.32 (0.11 to 0.74) | 0.13 (-0.48 to 0.75) | 9.72 (3.53 to 20.02) | 9.44 (3.57 to 19.38) | 0.02 (-0.38 to 0.43) |
| Israel | 0.83 (0.27 to 1.81) | 0.57 (0.18 to 1.28) | -1.51 (-2.31 to -0.71) | 14.36 (5.09 to 29.67) | 10.04 (3.68 to 20.97) | -1.47 (-2.26 to -0.66) |
| Italy | 0.16 (0.05 to 0.37) | 0.16 (0.05 to 0.36) | 0.17 (-0.24 to 0.58) | 3.9 (1.44 to 8.09) | 3.24 (1.2 to 6.84) | -0.64 (-0.91 to -0.36) |
| Jamaica | 0.53 (0.16 to 1.31) | 0.57 (0.16 to 1.42) | 0.38 (-1.83 to 2.63) | 9.87 (2.88 to 24.41) | 11.21 (3.13 to 28.01) | 0.57 (-1.48 to 2.66) |
| Japan | 0.44 (0.15 to 0.97) | 0.39 (0.13 to 0.88) | -0.34 (-0.74 to 0.07) | 7.9 (2.75 to 16.57) | 7.44 (2.66 to 15.65) | -0.34 (-0.71 to 0.03) |
| Jordan | 0.26 (0.07 to 0.7) | 0.53 (0.13 to 1.42) | 2.44 (1.86 to 3.03) | 5.03 (1.35 to 13.61) | 9.32 (2.35 to 24.87) | 2.08 (1.6 to 2.56) |
| Kazakhstan | 0.05 (0.01 to 0.14) | 0.13 (0.03 to 0.32) | 2.13 (0.76 to 3.51) | 3.61 (0.99 to 8.82) | 4.23 (1.22 to 9.9) | 0.23 (-0.36 to 0.83) |
| Kenya | 0.16 (0.04 to 0.43) | 0.37 (0.09 to 1) | 2.86 (2.78 to 2.95) | 2.91 (0.75 to 7.87) | 6.66 (1.69 to 17.74) | 2.73 (2.65 to 2.81) |
| Kiribati | 0.42 (0.1 to 1.17) | 0.84 (0.19 to 2.45) | 2.26 (2.12 to 2.4) | 8.19 (1.93 to 22.8) | 15.63 (3.56 to 45.88) | 2.11 (2.01 to 2.21) |
| Kuwait | 0.22 (0.06 to 0.56) | 0.35 (0.09 to 0.92) | 2.04 (1.03 to 3.05) | 4.39 (1.25 to 10.91) | 6.69 (1.71 to 17.24) | 1.87 (0.94 to 2.82) |
| Kyrgyzstan | 0.02 (0.01 to 0.06) | 0.08 (0.02 to 0.21) | 3.73 (2.4 to 5.08) | 2.3 (0.59 to 6.26) | 2.98 (0.82 to 7.66) | 0.59 (0.14 to 1.05) |
| Lao People's Democratic Republic | 0.15 (0.05 to 0.39) | 0.23 (0.06 to 0.59) | 1.37 (1.29 to 1.45) | 3.24 (0.97 to 8.05) | 4.57 (1.32 to 11.95) | 1.17 (1.07 to 1.27) |
| Latvia | 0.02 (0 to 0.05) | 0.06 (0.01 to 0.17) | 3.79 (3.15 to 4.43) | 1.52 (0.42 to 3.86) | 1.83 (0.5 to 4.73) | 0.48 (0.17 to 0.79) |
| Lebanon | 0.23 (0.06 to 0.65) | 0.23 (0.06 to 0.65) | -0.06 (-0.24 to 0.13) | 4.56 (1.11 to 12.31) | 4.29 (1.1 to 11.65) | -0.29 (-0.49 to -0.09) |
| Lesotho | 0.12 (0.03 to 0.32) | 0.25 (0.06 to 0.69) | 2.85 (2.45 to 3.26) | 3.19 (0.83 to 8.42) | 6.01 (1.57 to 15.27) | 2.22 (1.98 to 2.46) |
| Liberia | 0.09 (0.03 to 0.22) | 0.07 (0.02 to 0.18) | -0.79 (-1.07 to -0.5) | 1.81 (0.54 to 4.39) | 1.45 (0.46 to 3.57) | -0.81 (-1.07 to -0.54) |
| Libya | 0.27 (0.07 to 0.75) | 0.24 (0.06 to 0.64) | -0.53 (-1.01 to -0.05) | 5.72 (1.48 to 15.45) | 4.71 (1.19 to 12.49) | -0.75 (-1.21 to -0.29) |
| Lithuania | 0.02 (0 to 0.04) | 0.08 (0.02 to 0.19) | 5.3 (4.29 to 6.32) | 1.83 (0.5 to 4.58) | 3.05 (0.95 to 7.15) | 1.58 (1.14 to 2.01) |
| Luxembourg | 0.4 (0.11 to 0.95) | 0.18 (0.05 to 0.44) | -2.23 (-2.56 to -1.9) | 9.56 (2.92 to 21.25) | 4.07 (1.32 to 9.03) | -2.61 (-2.83 to -2.4) |
| Madagascar | 0.18 (0.05 to 0.5) | 0.18 (0.04 to 0.49) | -0.05 (-0.22 to 0.12) | 3.41 (0.89 to 9.5) | 3.23 (0.83 to 8.72) | -0.15 (-0.32 to 0.03) |
| Malawi | 0.19 (0.05 to 0.49) | 0.4 (0.1 to 1.08) | 2.68 (2.37 to 2.99) | 3.45 (0.91 to 8.97) | 7.17 (1.82 to 19.52) | 2.58 (2.28 to 2.89) |
| Malaysia | 0.25 (0.08 to 0.57) | 0.46 (0.15 to 1.07) | 2.02 (1.6 to 2.43) | 5.64 (1.94 to 12.45) | 9.81 (3.23 to 22.39) | 1.86 (1.46 to 2.26) |
| Maldives | 0.2 (0.05 to 0.53) | 0.15 (0.04 to 0.37) | -0.99 (-1.21 to -0.76) | 3.97 (1.1 to 10.4) | 2.75 (0.8 to 6.84) | -1.26 (-1.52 to -1) |
| Mali | 0.07 (0.02 to 0.18) | 0.12 (0.03 to 0.3) | 1.64 (1.53 to 1.75) | 1.39 (0.42 to 3.49) | 2.4 (0.71 to 5.97) | 1.72 (1.61 to 1.82) |
| Malta | 0.51 (0.17 to 1.16) | 0.32 (0.1 to 0.73) | -1.65 (-2.09 to -1.21) | 11.9 (4.13 to 25.13) | 7.45 (2.49 to 16.29) | -1.64 (-2.04 to -1.23) |
| Marshall Islands | 0.33 (0.08 to 0.93) | 1.13 (0.15 to 4.33) | 4.06 (3.92 to 4.2) | 6.51 (1.58 to 18.4) | 21.73 (3.06 to 82.78) | 3.99 (3.83 to 4.15) |
| Mauritania | 0.13 (0.04 to 0.34) | 0.24 (0.06 to 0.66) | 2.05 (1.88 to 2.23) | 2.59 (0.78 to 6.56) | 4.6 (1.25 to 12.34) | 1.91 (1.75 to 2.08) |
| Mauritius | 0.58 (0.14 to 1.54) | 2.51 (0.64 to 6.23) | 4.8 (3.96 to 5.65) | 11.73 (2.92 to 30.93) | 46.84 (11.87 to 115.55) | 4.52 (3.72 to 5.32) |
| Mexico | 1.05 (0.32 to 2.36) | 1.48 (0.47 to 3.2) | 1.11 (-0.12 to 2.35) | 19.49 (6.17 to 43.63) | 30.26 (9.77 to 63.66) | 1.75 (0.42 to 3.1) |
| Micronesia (Federated States of) | 0.54 (0.13 to 1.56) | 1.01 (0.26 to 2.83) | 2 (1.85 to 2.14) | 10.31 (2.52 to 29.09) | 18.72 (4.79 to 52.04) | 1.93 (1.83 to 2.03) |
| Monaco | 0.59 (0.2 to 1.32) | 0.79 (0.25 to 1.85) | 1.06 (0.92 to 1.21) | 15.84 (5.83 to 33.29) | 17.44 (6.16 to 38.62) | 0.36 (0.24 to 0.49) |
| Mongolia | 0.11 (0.03 to 0.31) | 0.11 (0.03 to 0.28) | -0.3 (-0.64 to 0.04) | 3.6 (0.92 to 9.37) | 3.39 (0.95 to 8.65) | -0.22 (-0.42 to -0.02) |
| Montenegro | 0.11 (0.03 to 0.27) | 0.1 (0.03 to 0.26) | 0.19 (-0.32 to 0.69) | 2.8 (0.82 to 6.63) | 2.17 (0.67 to 5.16) | -0.61 (-0.86 to -0.35) |
| Morocco | 0.08 (0.02 to 0.19) | 0.15 (0.05 to 0.34) | 2.18 (2.05 to 2.31) | 1.57 (0.55 to 3.68) | 2.82 (1.02 to 6.19) | 1.96 (1.85 to 2.06) |
| Mozambique | 0.08 (0.02 to 0.22) | 0.22 (0.06 to 0.59) | 3.31 (3.13 to 3.5) | 1.43 (0.41 to 3.82) | 3.76 (1.01 to 10.02) | 3.21 (3.07 to 3.34) |
| Myanmar | 0.06 (0.02 to 0.16) | 0.14 (0.04 to 0.35) | 2.54 (2.46 to 2.61) | 1.41 (0.42 to 3.37) | 2.78 (0.83 to 7.28) | 2.3 (2.23 to 2.37) |
| Namibia | 0.08 (0.02 to 0.21) | 0.07 (0.02 to 0.21) | 0.13 (-0.17 to 0.42) | 2.04 (0.59 to 5.25) | 1.74 (0.49 to 4.96) | -0.26 (-0.5 to -0.01) |
| Nauru | 1 (0.25 to 2.62) | 1.76 (0.38 to 5.13) | 2.01 (1.69 to 2.33) | 19.3 (4.85 to 50.18) | 34.43 (7.48 to 99.1) | 2.08 (1.76 to 2.4) |
| Nepal | 0.03 (0.01 to 0.08) | 0.06 (0.02 to 0.15) | 2.19 (2.05 to 2.34) | 0.76 (0.24 to 1.83) | 1.38 (0.41 to 3.46) | 1.87 (1.53 to 2.21) |
| Netherlands | 0.18 (0.06 to 0.41) | 0.3 (0.11 to 0.65) | 1.7 (1.32 to 2.09) | 5.02 (1.75 to 10.5) | 6.54 (2.45 to 13.4) | 0.9 (0.74 to 1.07) |
| New Zealand | 0.12 (0.04 to 0.28) | 0.14 (0.05 to 0.32) | 0.52 (-0.38 to 1.42) | 3.4 (1.16 to 7.32) | 3.91 (1.44 to 7.92) | 0.4 (-0.35 to 1.16) |
| Nicaragua | 0.16 (0.04 to 0.42) | 0.5 (0.13 to 1.18) | 3.85 (3.3 to 4.39) | 3.58 (1.02 to 9.07) | 10.83 (3.02 to 25.17) | 3.77 (3.27 to 4.27) |
| Niger | 0.04 (0.01 to 0.11) | 0.05 (0.01 to 0.13) | 0.59 (0.29 to 0.9) | 0.98 (0.3 to 2.4) | 1.18 (0.35 to 2.99) | 0.67 (0.43 to 0.91) |
| Nigeria | 0.05 (0.02 to 0.13) | 0.1 (0.03 to 0.25) | 2.18 (1.95 to 2.41) | 1.19 (0.4 to 2.71) | 2.25 (0.68 to 5.56) | 2.22 (2.12 to 2.32) |
| Niue | 0.64 (0.17 to 1.72) | 2.45 (0.53 to 6.88) | 4.44 (4.31 to 4.58) | 12.47 (3.2 to 33.19) | 45.11 (9.88 to 123.38) | 4.28 (4.16 to 4.41) |
| North Macedonia | 0.07 (0.02 to 0.19) | 0.11 (0.03 to 0.26) | 1.45 (1.09 to 1.81) | 2.4 (0.65 to 5.9) | 2.97 (0.87 to 6.76) | 0.73 (0.57 to 0.88) |
| Northern Mariana Islands | 3.4 (0.82 to 8.35) | 3.21 (0.84 to 8.27) | -0.32 (-0.57 to -0.07) | 60.52 (14.47 to 149.07) | 55.51 (14.56 to 142.95) | -0.31 (-0.45 to -0.18) |
| Norway | 0.06 (0.02 to 0.15) | 0.14 (0.05 to 0.31) | 2.43 (2.05 to 2.82) | 2.59 (0.96 to 5.31) | 3.75 (1.44 to 7.6) | 1.14 (0.83 to 1.46) |
| Oman | 0.11 (0.03 to 0.27) | 0.31 (0.08 to 0.82) | 3.41 (2.96 to 3.87) | 2.27 (0.65 to 5.81) | 5.9 (1.54 to 15.46) | 3.13 (2.67 to 3.58) |
| Pakistan | 0.05 (0.02 to 0.13) | 0.06 (0.02 to 0.16) | 0.55 (0.25 to 0.86) | 1.14 (0.35 to 2.66) | 1.33 (0.4 to 3.31) | 0.48 (0.18 to 0.79) |
| Palau | 0.82 (0.2 to 2.23) | 2.23 (0.52 to 6.16) | 3.32 (3.15 to 3.49) | 15.37 (3.64 to 41.07) | 39.67 (9.47 to 108.91) | 3.14 (2.99 to 3.29) |
| Palestine | 0.1 (0.03 to 0.25) | 0.07 (0.02 to 0.18) | -1.04 (-1.37 to -0.71) | 1.76 (0.49 to 4.5) | 1.31 (0.41 to 3.24) | -1.08 (-1.43 to -0.72) |
| Panama | 0.14 (0.04 to 0.36) | 0.31 (0.09 to 0.83) | 2.79 (1.88 to 3.7) | 3.52 (1.02 to 8.62) | 6.66 (1.85 to 17.4) | 2.22 (1.48 to 2.96) |
| Papua New Guinea | 0.09 (0.03 to 0.24) | 0.09 (0.03 to 0.22) | -0.05 (-0.23 to 0.13) | 2.07 (0.62 to 5.35) | 1.88 (0.58 to 4.65) | -0.25 (-0.44 to -0.05) |
| Paraguay | 0.36 (0.1 to 0.96) | 0.37 (0.1 to 0.99) | 0.54 (0.13 to 0.95) | 7.23 (2.11 to 19.2) | 7.14 (1.97 to 18.65) | 0.29 (0 to 0.59) |
| Peru | 0.45 (0.15 to 0.99) | 0.86 (0.28 to 1.9) | 2.19 (1.03 to 3.37) | 8.17 (2.72 to 17.97) | 16.5 (5.61 to 35.41) | 2.43 (1.47 to 3.4) |
| Philippines | 0.26 (0.09 to 0.58) | 0.56 (0.2 to 1.17) | 2.58 (2.17 to 2.99) | 4.76 (1.71 to 10.35) | 11.2 (4.06 to 22.83) | 2.95 (2.76 to 3.14) |
| Poland | 0.18 (0.06 to 0.4) | 0.11 (0.04 to 0.25) | -1.39 (-2.22 to -0.54) | 4.42 (1.53 to 9.19) | 3.31 (1.3 to 6.61) | -0.73 (-1.59 to 0.14) |
| Portugal | 0.28 (0.09 to 0.64) | 0.26 (0.08 to 0.67) | -0.19 (-0.99 to 0.6) | 5.42 (1.91 to 11.68) | 4.62 (1.54 to 10.96) | -1.11 (-2.5 to 0.3) |
| Puerto Rico | 0.69 (0.17 to 1.96) | 0.82 (0.21 to 2.18) | 0.51 (-0.06 to 1.08) | 12.99 (3.18 to 36.14) | 15.16 (3.9 to 40.77) | 0.55 (-0.02 to 1.12) |
| Qatar | 0.71 (0.18 to 1.88) | 2.03 (0.52 to 5.06) | 2.99 (2.16 to 3.84) | 13.39 (3.55 to 34.22) | 37.05 (9.69 to 90.54) | 3.02 (2.33 to 3.71) |
| Republic of Korea | 0.19 (0.06 to 0.48) | 0.2 (0.06 to 0.48) | -0.04 (-0.57 to 0.5) | 3.2 (0.99 to 7.72) | 3.3 (1.09 to 7.66) | 0.06 (-0.26 to 0.38) |
| Republic of Moldova | 0 (0 to 0.01) | 0.01 (0 to 0.02) | 1.97 (0.49 to 3.48) | 0.6 (0.18 to 1.56) | 0.64 (0.18 to 1.68) | -0.03 (-0.33 to 0.28) |
| Romania | 0.05 (0.02 to 0.1) | 0.13 (0.05 to 0.27) | 4.08 (1.96 to 6.25) | 2.27 (0.78 to 4.63) | 5.04 (1.93 to 9.88) | 2.73 (2.29 to 3.16) |
| Russian Federation | 0.03 (0.01 to 0.06) | 0.07 (0.02 to 0.16) | 3.25 (2.34 to 4.17) | 1.28 (0.45 to 2.73) | 1.93 (0.72 to 4) | 1.16 (0.78 to 1.53) |
| Rwanda | 0.14 (0.04 to 0.37) | 0.17 (0.04 to 0.47) | 0.7 (0.58 to 0.83) | 2.58 (0.68 to 6.81) | 3.03 (0.76 to 8.3) | 0.56 (0.43 to 0.7) |
| Saint Kitts and Nevis | 0.62 (0.18 to 1.54) | 0.7 (0.21 to 1.68) | 0.61 (-0.12 to 1.35) | 11.43 (3.4 to 28.37) | 12.35 (3.72 to 29.57) | 0.45 (-0.17 to 1.08) |
| Saint Lucia | 0.43 (0.14 to 1.02) | 0.47 (0.14 to 1.14) | 0.25 (-0.23 to 0.74) | 7.85 (2.5 to 18.46) | 8.18 (2.47 to 20.1) | 0.23 (-0.22 to 0.68) |
| Saint Vincent and the Grenadines | 0.28 (0.09 to 0.72) | 0.72 (0.22 to 1.7) | 2.99 (2.33 to 3.66) | 5.24 (1.62 to 13.17) | 12.56 (3.91 to 29.75) | 3.15 (2.27 to 4.03) |
| Samoa | 0.62 (0.16 to 1.69) | 1.59 (0.4 to 4.25) | 3.1 (2.99 to 3.2) | 12.06 (3.17 to 33.34) | 30.01 (7.69 to 79.84) | 2.98 (2.87 to 3.1) |
| San Marino | 0.26 (0.08 to 0.61) | 0.12 (0.03 to 0.3) | -2.55 (-3.04 to -2.05) | 7.13 (2.26 to 16.08) | 4.2 (1.26 to 9.47) | -1.73 (-1.93 to -1.53) |
| Sao Tome and Principe | 0.13 (0.04 to 0.34) | 0.21 (0.06 to 0.56) | 1.45 (1.31 to 1.6) | 2.47 (0.7 to 6.17) | 3.9 (1.11 to 10.25) | 1.52 (1.38 to 1.66) |
| Saudi Arabia | 1.02 (0.34 to 2.33) | 2.37 (0.83 to 5.09) | 2.78 (2.64 to 2.91) | 21.1 (7.34 to 46.68) | 45.12 (16.44 to 94.69) | 2.52 (2.4 to 2.64) |
| Senegal | 0.05 (0.02 to 0.12) | 0.06 (0.02 to 0.14) | -0.02 (-0.3 to 0.26) | 1.07 (0.41 to 2.15) | 1.09 (0.34 to 2.69) | -0.12 (-0.4 to 0.16) |
| Serbia | 0.22 (0.06 to 0.54) | 0.14 (0.05 to 0.32) | -1.25 (-1.46 to -1.03) | 4.53 (1.32 to 10.82) | 3.06 (1 to 6.54) | -1.26 (-1.48 to -1.04) |
| Seychelles | 0.14 (0.04 to 0.36) | 0.26 (0.07 to 0.69) | 1.66 (0.56 to 2.77) | 3.3 (1 to 8) | 4.75 (1.31 to 12.87) | 1.36 (0.36 to 2.37) |
| Sierra Leone | 0.04 (0.01 to 0.09) | 0.04 (0.01 to 0.09) | 0.03 (-0.17 to 0.23) | 0.73 (0.22 to 1.72) | 0.78 (0.24 to 1.92) | 0.16 (0 to 0.32) |
| Singapore | 0.24 (0.07 to 0.59) | 0.37 (0.11 to 0.88) | 1.31 (-0.08 to 2.72) | 4.34 (1.35 to 10.14) | 6.59 (2.13 to 15.01) | 1.22 (-0.02 to 2.47) |
| Slovakia | 0.16 (0.05 to 0.37) | 0.12 (0.03 to 0.28) | -1.11 (-1.44 to -0.77) | 4.27 (1.5 to 9.38) | 3.04 (0.96 to 6.82) | -1.24 (-1.63 to -0.84) |
| Slovenia | 0.07 (0.02 to 0.16) | 0.09 (0.02 to 0.24) | 1.03 (0.44 to 1.62) | 2.38 (0.74 to 5.21) | 2.61 (0.78 to 6.12) | 0.19 (-0.22 to 0.6) |
| Solomon Islands | 0.2 (0.05 to 0.52) | 0.19 (0.06 to 0.47) | -0.29 (-0.74 to 0.16) | 4 (1.09 to 10.26) | 3.49 (1.07 to 8.55) | -0.39 (-0.97 to 0.19) |
| Somalia | 0.18 (0.05 to 0.47) | 0.17 (0.04 to 0.47) | -0.15 (-0.22 to -0.08) | 3.42 (0.93 to 8.92) | 3.19 (0.83 to 8.95) | -0.17 (-0.22 to -0.12) |
| South Africa | 0.17 (0.05 to 0.39) | 0.26 (0.09 to 0.55) | 1.34 (0.31 to 2.38) | 4.31 (1.59 to 8.94) | 5.79 (2.29 to 11.45) | 0.95 (0.35 to 1.57) |
| South Sudan | 0.48 (0.11 to 1.39) | 0.76 (0.18 to 2.11) | 1.56 (1.51 to 1.62) | 8.95 (2.08 to 25.68) | 13.92 (3.42 to 38.95) | 1.51 (1.44 to 1.59) |
| Spain | 0.32 (0.1 to 0.74) | 0.23 (0.07 to 0.54) | -1.38 (-1.93 to -0.82) | 6.46 (2.25 to 14.11) | 4.85 (1.75 to 10.66) | -1.03 (-1.39 to -0.68) |
| Sri Lanka | 0.14 (0.04 to 0.36) | 0.22 (0.06 to 0.59) | 1.9 (1.21 to 2.59) | 2.77 (0.81 to 6.99) | 4.42 (1.17 to 11.42) | 1.9 (1.23 to 2.57) |
| Sudan | 0.04 (0.01 to 0.12) | 0.06 (0.02 to 0.17) | 1.51 (1.22 to 1.81) | 1 (0.29 to 2.77) | 1.34 (0.4 to 3.46) | 1.1 (0.84 to 1.36) |
| Suriname | 0.58 (0.16 to 1.48) | 0.75 (0.21 to 1.84) | 0.73 (0.06 to 1.41) | 11.86 (3.23 to 30.12) | 14.41 (3.98 to 35.66) | 0.49 (-0.34 to 1.32) |
| Sweden | 0.08 (0.02 to 0.18) | 0.22 (0.07 to 0.52) | 3.7 (3.11 to 4.3) | 2.42 (0.84 to 5.06) | 4.18 (1.44 to 9.15) | 1.96 (1.63 to 2.29) |
| Switzerland | 0.11 (0.03 to 0.26) | 0.13 (0.04 to 0.29) | 0.45 (-0.01 to 0.91) | 3.21 (1.13 to 6.92) | 2.91 (1.09 to 6.12) | -0.37 (-0.69 to -0.05) |
| Syrian Arab Republic | 0.34 (0.08 to 0.94) | 0.25 (0.07 to 0.66) | -0.92 (-1.59 to -0.25) | 6.59 (1.62 to 18.47) | 4.67 (1.31 to 12.41) | -1.02 (-1.79 to -0.24) |
| Taiwan (Province of China) | 0.57 (0.19 to 1.33) | 0.56 (0.17 to 1.31) | -0.13 (-0.74 to 0.49) | 9.52 (3.25 to 21.23) | 10.43 (3.35 to 23.35) | 0.23 (-0.12 to 0.59) |
| Tajikistan | 0 (0 to 0.01) | 0 (0 to 0.01) | -0.13 (-0.83 to 0.57) | 0.41 (0.12 to 0.99) | 0.32 (0.1 to 0.81) | -0.81 (-1.02 to -0.6) |
| Thailand | 0.27 (0.08 to 0.65) | 0.65 (0.22 to 1.41) | 2.98 (2.72 to 3.24) | 5.35 (1.77 to 12.06) | 13.91 (4.96 to 29.27) | 3.19 (2.99 to 3.4) |
| Timor-Leste | 0.08 (0.02 to 0.21) | 0.08 (0.02 to 0.2) | -0.12 (-0.22 to -0.03) | 1.67 (0.48 to 4.11) | 1.65 (0.45 to 3.99) | -0.04 (-0.13 to 0.04) |
| Togo | 0.05 (0.01 to 0.12) | 0.09 (0.03 to 0.23) | 2.12 (1.99 to 2.26) | 0.99 (0.31 to 2.39) | 1.85 (0.54 to 4.5) | 2.07 (1.94 to 2.2) |
| Tokelau | 0.3 (0.07 to 0.93) | 0.98 (0.24 to 2.65) | 3.92 (3.76 to 4.08) | 5.79 (1.43 to 17.55) | 17.4 (4.49 to 45.59) | 3.67 (3.51 to 3.83) |
| Tonga | 0.18 (0.04 to 0.52) | 0.45 (0.09 to 1.43) | 3.15 (2.92 to 3.38) | 3.83 (0.91 to 11.06) | 8.97 (1.87 to 27.86) | 2.82 (2.66 to 2.98) |
| Trinidad and Tobago | 0.49 (0.15 to 1.15) | 1.1 (0.31 to 2.7) | 3.01 (1.29 to 4.76) | 9.24 (2.76 to 21.39) | 21.59 (6 to 53.9) | 3.17 (1.41 to 4.96) |
| Tunisia | 0.22 (0.05 to 0.6) | 0.36 (0.09 to 0.93) | 1.6 (1.4 to 1.8) | 4.58 (1.16 to 11.95) | 6.72 (1.82 to 16.76) | 1.24 (1.04 to 1.44) |
| Turkey | 0.64 (0.19 to 1.51) | 0.62 (0.19 to 1.52) | -0.17 (-0.55 to 0.21) | 12.24 (3.96 to 27.74) | 11.69 (3.9 to 27.01) | -0.25 (-0.61 to 0.11) |
| Turkmenistan | 0.06 (0.02 to 0.17) | 0.23 (0.06 to 0.58) | 4.41 (2.77 to 6.08) | 3.11 (0.79 to 8.05) | 7.3 (1.97 to 17.47) | 3.13 (2.54 to 3.72) |
| Tuvalu | 0.31 (0.09 to 0.84) | 0.72 (0.18 to 2.01) | 2.79 (2.69 to 2.88) | 6.28 (1.69 to 16.92) | 13.72 (3.43 to 38.31) | 2.61 (2.51 to 2.71) |
| Uganda | 0.14 (0.04 to 0.36) | 0.22 (0.06 to 0.58) | 1.54 (1.47 to 1.62) | 2.59 (0.71 to 6.55) | 3.97 (1.09 to 10.33) | 1.39 (1.32 to 1.46) |
| Ukraine | 0 (0 to 0) | 0.01 (0 to 0.01) | 9.72 (6.68 to 12.84) | 0.53 (0.17 to 1.24) | 0.57 (0.2 to 1.25) | 0.17 (-0.11 to 0.46) |
| United Arab Emirates | 0.69 (0.22 to 1.52) | 1.14 (0.32 to 2.77) | 2.81 (1.15 to 4.49) | 15.21 (5.19 to 31.98) | 21.24 (6.22 to 49.99) | 2.15 (0.71 to 3.61) |
| United Kingdom | 0.11 (0.04 to 0.25) | 0.13 (0.04 to 0.28) | 0.88 (0.29 to 1.47) | 3.88 (1.38 to 8.1) | 3.86 (1.43 to 8.03) | 0.17 (-0.1 to 0.43) |
| United Republic of Tanzania | 0.2 (0.06 to 0.55) | 0.21 (0.05 to 0.57) | 0.13 (0.04 to 0.21) | 4.22 (1.16 to 10.88) | 4.12 (1.12 to 10.93) | -0.03 (-0.09 to 0.04) |
| United States of America | 0.41 (0.14 to 0.89) | 1.31 (0.53 to 2.48) | 3.96 (3.47 to 4.45) | 10.3 (3.83 to 20.85) | 26.09 (10.74 to 48.42) | 3.11 (2.66 to 3.55) |
| United States Virgin Islands | 0.39 (0.11 to 1) | 0.62 (0.16 to 1.58) | 1.44 (1.03 to 1.85) | 7.77 (2.19 to 19.81) | 11.51 (3.01 to 29.44) | 1.16 (0.61 to 1.71) |
| Uruguay | 0.45 (0.14 to 0.98) | 0.62 (0.19 to 1.42) | 1.27 (0.39 to 2.15) | 8.78 (2.62 to 19.26) | 11.48 (3.74 to 25.2) | 0.73 (-0.07 to 1.54) |
| Uzbekistan | 0.04 (0.01 to 0.14) | 0.07 (0.02 to 0.18) | 1.52 (-1.11 to 4.23) | 2.24 (0.54 to 6.25) | 2.36 (0.68 to 5.69) | 0.37 (-1.74 to 2.52) |
| Vanuatu | 0.37 (0.1 to 1.04) | 0.45 (0.13 to 1.21) | 0.66 (0.52 to 0.8) | 7.14 (1.92 to 19.92) | 8.39 (2.37 to 22.9) | 0.53 (0.32 to 0.73) |
| Venezuela (Bolivarian Republic of) | 0.31 (0.1 to 0.68) | 0.5 (0.17 to 1.15) | 1.83 (1.15 to 2.51) | 7.5 (2.56 to 16.03) | 10.39 (3.66 to 22.87) | 1.25 (0.62 to 1.87) |
| Viet Nam | 0.05 (0.02 to 0.11) | 0.12 (0.04 to 0.28) | 3.29 (3.12 to 3.45) | 0.9 (0.31 to 1.97) | 2.22 (0.75 to 4.76) | 3.17 (3.01 to 3.32) |
| Yemen | 0.06 (0.02 to 0.16) | 0.03 (0.01 to 0.09) | -1.64 (-1.77 to -1.52) | 1.26 (0.35 to 3.31) | 0.74 (0.21 to 1.88) | -1.75 (-1.88 to -1.62) |
| Zambia | 0.23 (0.06 to 0.58) | 0.24 (0.07 to 0.64) | 0.36 (0.19 to 0.53) | 4.38 (1.12 to 11.27) | 4.64 (1.31 to 12.18) | 0.26 (0.13 to 0.39) |
| Zimbabwe | 0.08 (0.02 to 0.21) | 0.09 (0.02 to 0.24) | 0.3 (-0.32 to 0.93) | 2.13 (0.61 to 5.13) | 2.06 (0.59 to 5.41) | 0.21 (-0.24 to 0.67) |

Abbreviations: ASPR, age-standardized prevalence rate; ASMR, age-standardized death rate; ASDR, age-standardized disability-adjusted life years rate; SDI, sociodemographic index; UI, uncertainty interval; AAPC, average annual percent change; CI, confidence interval.

**Supplementary Table 2.** Changes in deaths, and DALYs rate of chronic kidney disease due to diabetes mellitus type 2 attributable to diet high in sugar-sweetened beverages among elderly according to population-level determinants from 1990 to 2021 globally and 5 SDI regions.

| location | measure | Overll difference | Aging | Population | Epidemiological change |
| --- | --- | --- | --- | --- | --- |
| Low SDI | Deaths | 32.47 (131.65%) | 1.47 (5.98%) | 30.57 (123.93%) | 0.43 (1.74%) |
| Low SDI | DALYs (Disability-Adjusted Life Years) | 706.4 (120.7%) | 7.95 (1.36%) | 708.78 (121.1%) | -10.32 (-1.76%) |
| Low-middle SDI | Deaths | 225.98 (359.97%) | 7.96 (12.68%) | 131.06 (208.76%) | 86.97 (138.53%) |
| Low-middle SDI | DALYs (Disability-Adjusted Life Years) | 5065.76 (331.73%) | 53.04 (3.47%) | 3071.85 (201.16%) | 1940.87 (127.1%) |
| Global | Deaths | 2966.64 (346.31%) | 268.65 (31.36%) | 1574.86 (183.84%) | 1123.13 (131.11%) |
| Global | DALYs (Disability-Adjusted Life Years) | 57039.28 (282.84%) | 2265.56 (11.23%) | 33822.23 (167.72%) | 20951.48 (103.89%) |
| High-middle SDI | Deaths | 394.76 (214.71%) | 62.63 (34.06%) | 241.49 (131.34%) | 90.64 (49.3%) |
| High-middle SDI | DALYs (Disability-Adjusted Life Years) | 7560.05 (175.16%) | 562.29 (13.03%) | 5259.39 (121.86%) | 1738.37 (40.28%) |
| High SDI | Deaths | 1383.4 (338.52%) | 151.18 (36.99%) | 595.12 (145.63%) | 637.1 (155.9%) |
| High SDI | DALYs (Disability-Adjusted Life Years) | 24051.1 (247.6%) | 1162.65 (11.97%) | 12235.44 (125.96%) | 10653 (109.67%) |
| Middle SDI | Deaths | 927.93 (528.26%) | 64.89 (36.94%) | 501.53 (285.51%) | 361.51 (205.8%) |
| Middle SDI | DALYs (Disability-Adjusted Life Years) | 19616.22 (490.57%) | 470.08 (11.76%) | 11019.18 (275.57%) | 8126.96 (203.24%) |

Abbreviations: DALYs, disability-adjusted life years rate; SDI, sociodemographic index.

**Supplementary Fig. 1** Association between age-standardized deaths rate of chronic kidney disease due to diabetes mellitus type 2 attributable to diet high in sugar-sweetened beverages among elderly and 204 countries.


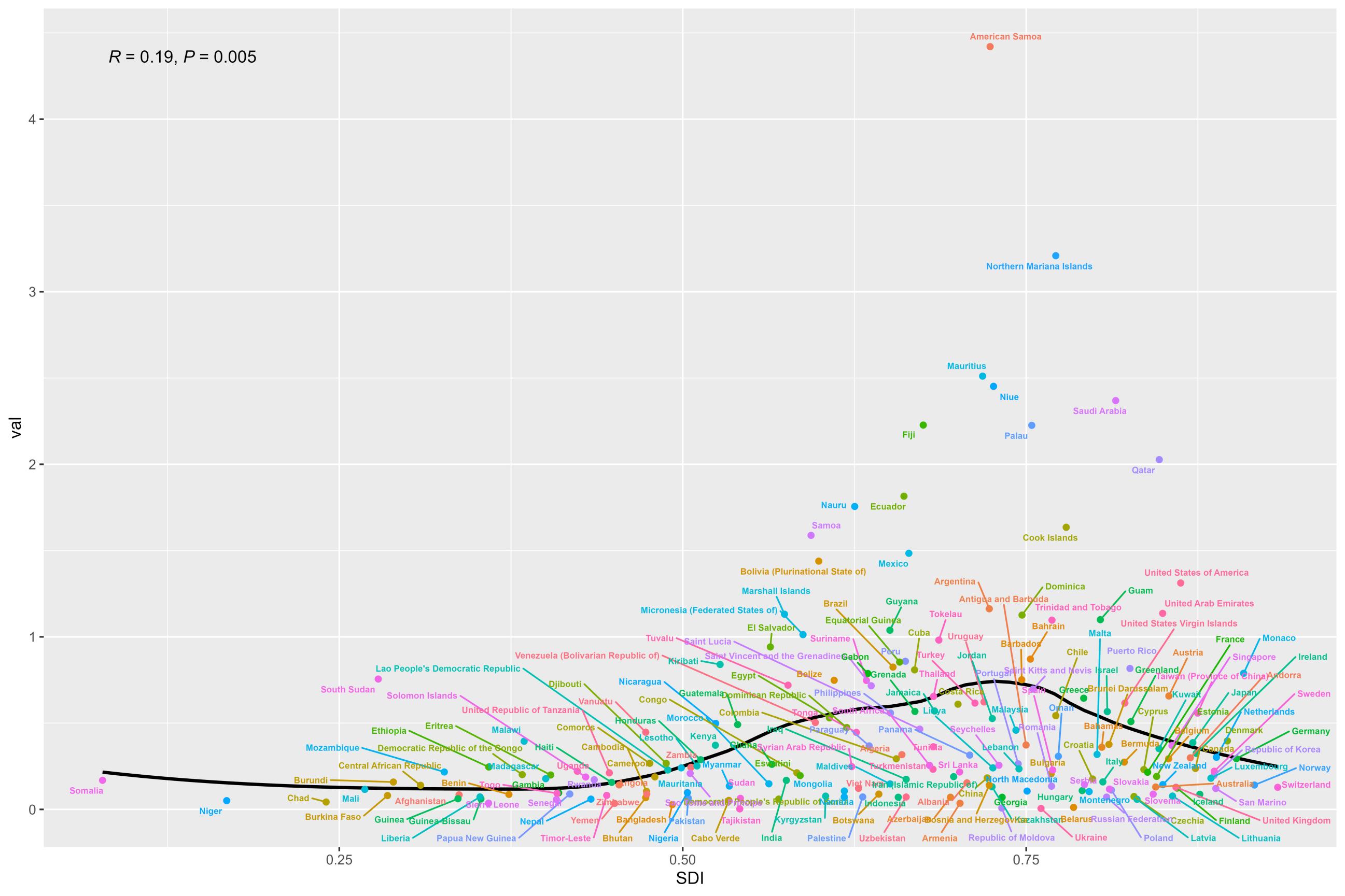


**Supplementary Fig. 2** Association between age-standardized DALYs rate of chronic kidney disease due to diabetes mellitus type 2 attributable to diet high in sugar-sweetened beverages among elderly and 204 countries.


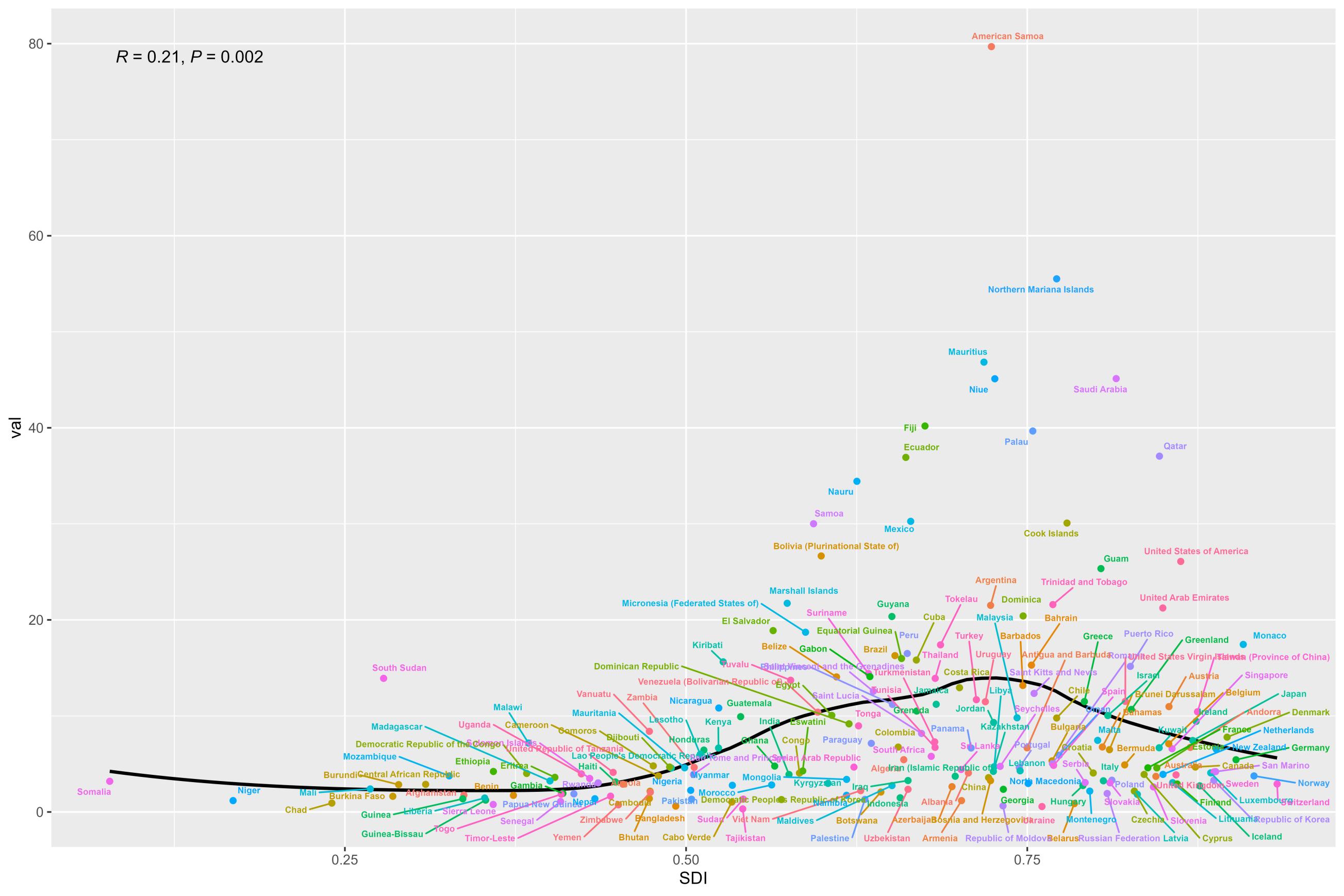


**Supplementary Fig. 3** Association between age-standardized deaths rate of chronic kidney disease due to diabetes mellitus type 2 attributable to diet high in sugar-sweetened beverages among elderly and 21 regions.


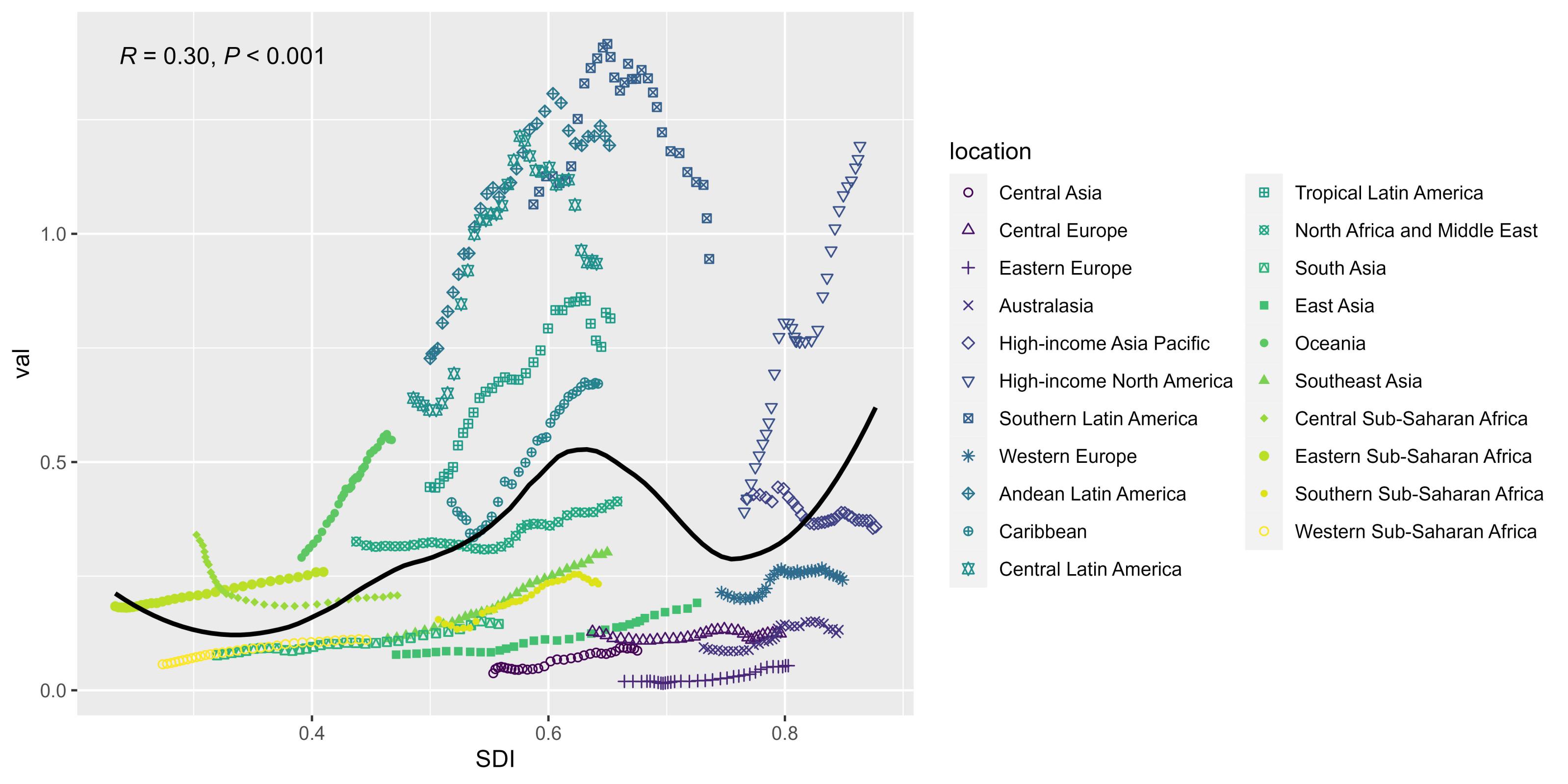


**Supplementary Fig. 4** Association between age-standardized DALYs rate of chronic kidney disease due to diabetes mellitus type 2 attributable to diet high in sugar-sweetened beverages among elderly and 21 regions.

**
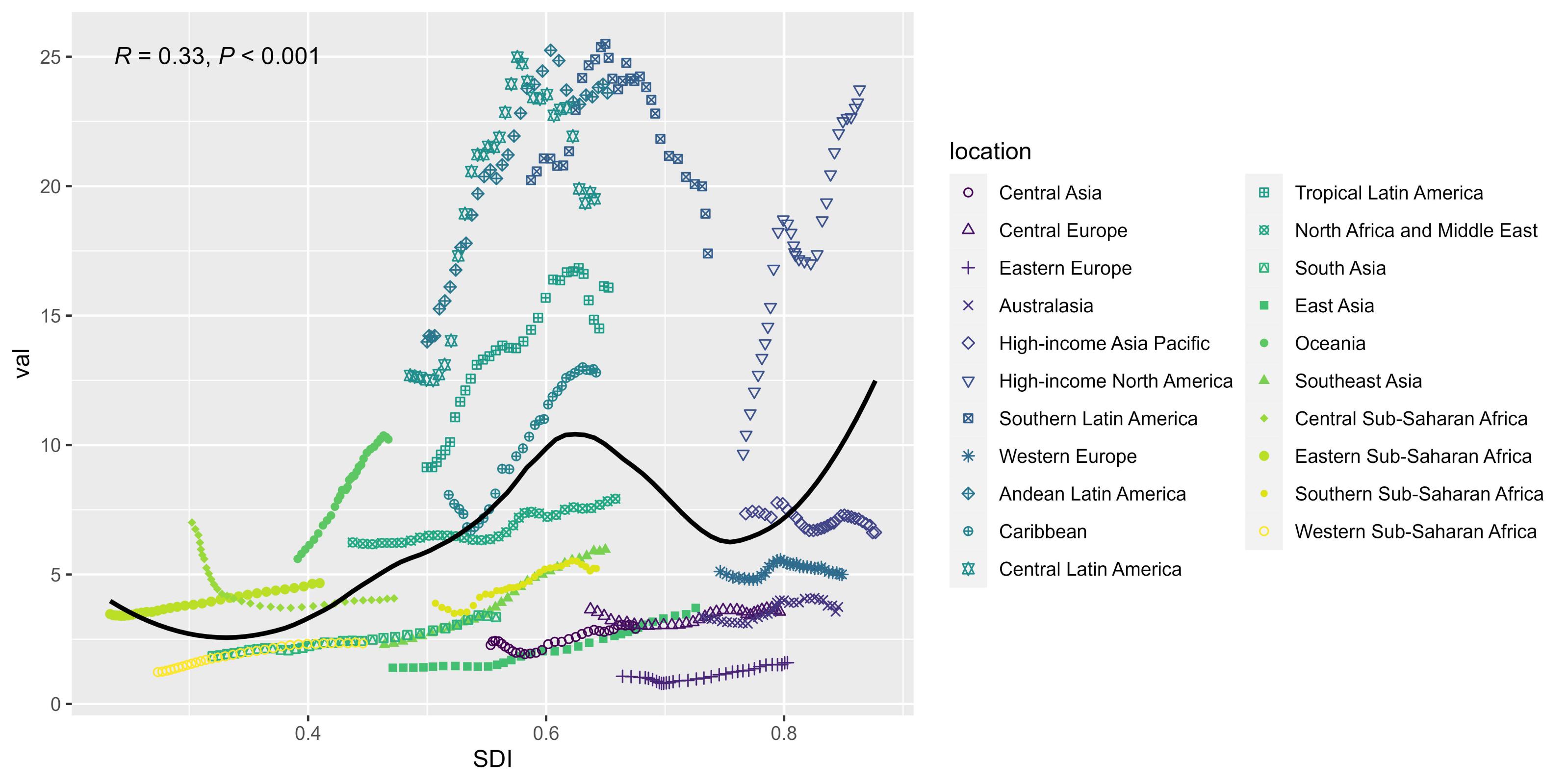
**
