# Supplementary material for: The global burden of chronic kidney disease due to diabetes mellitus type 2 attributable to diet high in sugar-sweetened beverages among the elderly: a comprehensive analysis from 1990 to 2021
Source: Front Nutr. 2025 Jun 19;12:1615351. doi: 10.3389/fnut.2025.1615351 (PMC12221943; doi:10.3389/fnut.2025.1615351)
Supplement: Supplementary file 2 [file Table_2.DOCX]

**Supplemental Methods**

**1. GBD data processing and modeling process.**

GBD 2021 compiled data sources through data identification and extraction. By identifying from systematic reviews, government and international organisation websites, published reports, primary data sources and contributions of datasets by GBD collaborators, the GBD study combined a variety of data sources including censuses, household surveys, civil registration and vital statistics, disease registries, health service use, air pollution monitors, satellite imaging, disease notifications and other sources, among which each of them was given a unique identifier and included in the Global Health Data Exchange (GHDx) (1). The systematic review in GBD 2021 mainly included three approaches: (1) electronic searches in databases, (2) grey literature searches and (3) consultation from experts. Garbage code redistribution and noise reduction data, together with small sample size were excluded. Data sources used in estimating the burden of chronic kidney disease due to diabetes mellitus type 2 attributable to diet high in sugar-sweetened beverages across different countries and territories worldwide can be found in the GBD 2021 data input source tool (<http://ghdx.healthdata.org/gbd-2021/datainput-sources>) (1, 2).

GBD 2021 corrected bias and adjusted data for further modelling process. The collected data were modelled by spatiotemporal Gaussian process regression to allow for smoothing over age, time and location in locations lacking complete datasets. The Meta-Regression with Bayesian priors, Regularisation and Trimming (MR-BRT) programme was adopted to adjust data bias for alternative case definitions and study methods. There are three types of age splitting and sex splitting: (1) Estimates were split by age and sex, where possible. Nevertheless, age-­ specific estimates would be calculated based on the reported sex ratio and uncertainty bounds when the incidence data were reported for specific age groups without separating by sex, or by sex for large age intervals; (2) The remaining individual sex estimates were performed with MR-BRT. MR­BRT network meta-analysis estimated the pooled sex ratios and uncertainty bounds, which were then adopted to split combined sex estimates and (3) Researches presenting prevalence estimates for age groups of 25+ years were split into 5-­year age groups using the age pattern generated by DisMod-MR 2.1 (1, 2).

GBD 2021 modelled the epidemiology of chronic kidney disease due to diabetes mellitus type 2 attributable to diet high in sugar-sweetened beverages using a meta-regression tool based on Bayesian model framework, namely, DisMod-MR 2.1. The Bayesian approach serves as an interpretation of statistical probability, where existing data are used to inform the probability of a given hypothesis. A meta-regression can be regarded as an extension of a meta-analysis whereby data from different sources are pooled into a weighted average adjusting for heterogeneities. DisMod-MR 2.1 applied a negative-binomial model of disease incidence, prevalence, remission and case-fatality rates, and fitted models with a randomised Markov-Chain Monte Carlo algorithm. The steps of modelling process in DisMod-MR 2.1 are as follows: (1) It pooled heterogeneous raw data for each parameter and adjusted data for methodological distinctions. If the data were insufficient to indicate an age-pattern variation, DisMod-MR 2.1 may impose a common age pattern according to assessment of age-specific input data for the disease; (2) It checked data on incidence, prevalence, duration, remission and mortality risk for internal consistency; (3) It simultaneously integrated the input data from all parameters plus to the outputs from previous steps to obtain internally consistent epidemiological estimates, carrying forward uncertainty from primary data sources; (4) Even for countries with little or no primary data source, this model could produce estimates based on information from the available data, and this process allowed for estimates of chronic kidney disease due to diabetes mellitus type 2 attributable to diet high in sugar-sweetened beverages burden in all countries worldwide (1, 2).

**2. Definition of DALYs**

Disability-adjusted life years (DALYs) are defined as the sum of years lost due to premature death (YLLs) and years lived with disability (YLDs) (1, 4).

**2.1 Calculation methods for DALYs**

To estimate DALYs, GBD 2021 started by estimating cause‐specific mortality and non‐fatal health loss. For each year for which YLDs have been estimated, GBD 2021 computed DALYs by adding YLLs and YLDs for each age-sex-location. Uncertainty in YLLs was assumed to be independent of uncertainty in YLDs. GBD 2021 calculated 1000 draws for DALYs by summing the first draw of the 1000 draws for YLLs and YLDs and then repeating for each subsequent draw. 95% UIs were computed by using the 25th and 975th ordered draw of the DALY uncertainty distribution. GBD 2021 calculated DALYs as the sum of YLLs and YLDs for each cause, location, age group, sex, and year. For more information, please refer to the following figure (1).


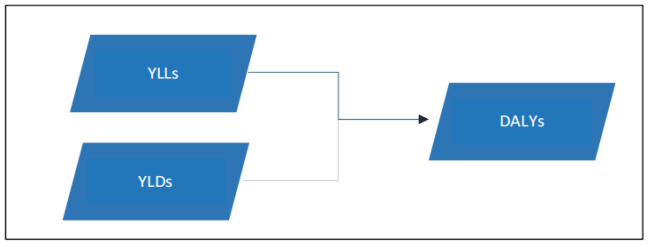


**2.2 YLLs**

The YLL is a metric that is computed by multiplying the number of estimated deaths by the standard life expectancy at age of death. The metric therefore highlights premature deaths by applying a larger weight to deaths that occur in younger age groups. The core equation can be written as follows (1):


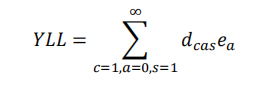


**2.3 YLDs**

YLD was computed by sequela as prevalence multiplied by the DW for the health state associated with that sequela (1).


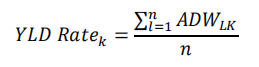


**2.4 Disability Weights (DWs)**

DWs are measured on a scale from 0 to 1; 0 implies a state equivalent to full health, and 1, a state equivalent to death. The formula for the cumulative DW is one minus the multiplicative sum of one minus each DW present (1):


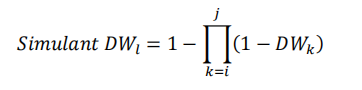


Where:

DW_k_ is the DW for the kth disease sequela that the simulant l has acquired.

Once the simulant DW is computed, the DW attributable to each sequela for the simulant is calculated by using the following formula:


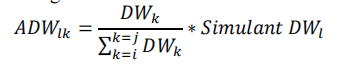


Where:

ADW_lk_ is the attributable DW for disease sequela k in simulant l DW_k_ is the DW for disease sequela k.

Simulant DW_l_ is the DW for simulant l from the combination of all sequelae that they have acquired.

This formula apportions the overall simulant DW to each condition in proportion to the DW of each condition in isolation.

Finally, YLDs per capita in an age-sex-country-year are computed by taking the sum of the attributable DWs for a disease sequela across simulants. The actual number of YLDs from disease sequela k in an age-sex-location-year is then computed as the YLD rate k times the appropriate age-sex-location-year population. GBD 2021 determined the disability weights for each sequela from the GBD disability weight survey (1).

**3. Joinpoint regression analysis**

Time trend analysis is an important component of epidemiological research. Traditional regression models primarily fit and evaluate the overall trend of disease distribution within the study period from a global perspective, failing to capture local variation characteristics. In 1998, Kim et al. first proposed the Joinpoint regression model. The core idea of this model is to establish segmented regression based on the temporal characteristics of disease distribution. By dividing the study time into different intervals through several Joinpoints, the trend in each interval is fitted and optimized, allowing for a more detailed assessment of the specific disease change characteristics within different intervals of the overall time range (5).

The Joinpoint regression model, developed by the Division of Cancer Control and Population Sciences at the National Cancer Institute of the United States, has been widely applied in the field of trend studies on disease incidence and mortality rates.

(I) Model Introduction

The Joinpoint regression model includes two types: the linear model (y = xb) and the logarithmic linear model (ln y = xb). If the dependent variable follows a normal distribution (or approximately normal distribution) and the sample size is large (usually greater than 100), the linear model is preferred. For example, when the dependent variable is continuous variables like height, weight, etc. If the dependent variable follows an exponential distribution or a Poisson distribution, the logarithmic linear model is more suitable. For instance, when the dependent variable represents epidemiological data based on populations such as incidence rates, number of cases, etc. When analyzing trends in the incidence, prevalence, mortality rates, and DALYs rates of thalassemia based on population data, the logarithmic linear model is generally chosen (5).

(II) Modeling Method

The grid search method (GSM) is the default modeling approach used by Joinpoint. GSM divides the study data into a grid, with each grid intersection corresponding to a planned scenario. Then, within the specified intervals, it computes performance metrics for the corresponding equations at each point using a fixed step size to determine the optimal function. In essence, the Joinpoint model uses the GSM to establish all possible segment function Joinpoints (i.e., Joinpoints) and calculates the sum of squares errors (SSE) and mean squared errors (MSE) for each possible scenario. It selects the grid point with the smallest MSE as the Joinpoint for the segment function and fits the equation parameters such as β_0_, β_1_, δ_1_, ..., δ_k_ based on the selected Joinpoints and interval functions (6).

(III) Model Optimization

Monte Carlo permutation test is the default model optimization method in Joinpoint software. Before modeling, it is necessary to set the range of the number of Joinpoints k as k ∈ (MIN，MAX) , where MIN represents the minimum number of Joinpoints, which is usually set to 0; MAX represents the maximum number of Joinpoints. Each permutation test checks the null hypothesis H_0_: the number of Joinpoints is k = k_a_, and the alternative hypothesis H_1_: the number of Joinpoints is k = k_b_. The permutation test starts from k_a_=MIN and k_b_=MAX. If H_0_ is rejected, k is set to k_a_ + 1 for further testing; if H_0_ is not rejected, k is set to k_b_ - 1 for another test, until k_a_ = k_b_, which means k = k_a_ = k_b_ is the preferred number of Joinpoints selected by the permutation test, and the corresponding model is the optimal model (7).

(IV) Index Calculation

Annual percent change (APC) and average annual percent change (AAPC) along with their 95% confidence intervals (CI) are the primary outcome indicators of the Joinpoint model. As the name suggests, APC represents the average annual percentage change of the dependent variable. For example, in a logarithmic linear model ln (y) =β_0_ + β_1_ x，where y represents the incidence rate and x represents the year of incidence, the formula for calculating APC in the fitted model can be derived as:


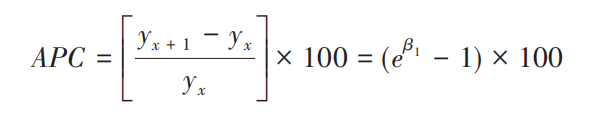


The lower and upper limits of the 100(1-α) % confidence interval are respectively:


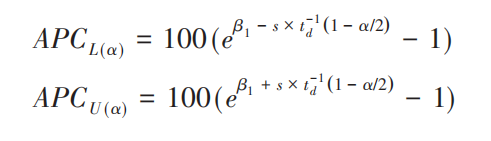


In the above formula, β_1_ represents the regression coefficient, s represents the standard error of β_1_, d represents the degrees of freedom, and t_d_(q) is the value corresponding to the qth percentile of the t-distribution with d degrees of freedom (such as 95%).

The APC is used to evaluate the internal trend of each independent interval of a segmented function or the overall trend with no connecting points. When it comes to assessing the overall average change trend encompassing multiple intervals, the AAPC is required. The parameter calculation method of AAPC involves weighted calculation of the regression coefficients of each interval based on the width w of the segment intervals. Its formula is as follows:


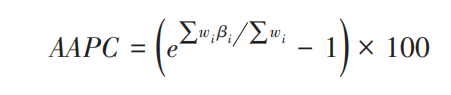


The lower and upper limits of the 100(1-α) % confidence interval are respectively:


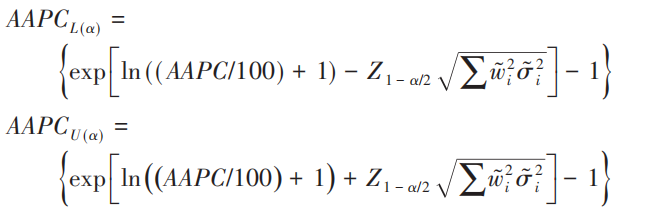


In the above formula, w_i_ represents the width of each segment function interval (i.e., the number of years included in the interval), β_i_ denotes the regression coefficient corresponding to each interval, σ^2^i is the variance of β_i_, and Z_α_ represents the corresponding value of the α percentile in the normal distribution (7).

(V) Software Download

To download Joinpoint software, we visited the website of the National Cancer Institute (https://surveillance.cancer.gov/Joinpoint/download), registered, andsubmitted our application information. Software citation: Joinpoint Regression Program, Version 4.9.1.0 - April 2022; Statistical Methodology and Applications Branch, Surveillance Research Program, National Cancer Institute.

**4.Calculated age-standardised rates (ASRs) per 100,000 people**

We calculated age-standardised rates (ASRs) per 100,000 people of elderly individuals from 60 to 95+ years, according to the formula (8):


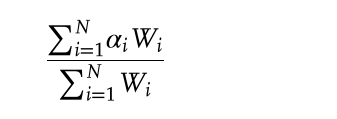


In the equation, αi denotes the age-specific rate in the ith age group, while Wi signifies the count of individuals within the same age group as per the GBD 2021 standard population. N is the total number of age groups. The 95% confidence interval (CI) was determined by “ageadjust.direct” function of package “epitools” within R software (9).

**7. Decomposition analysis:**

We first used the decomposition methodology of Das Gupta (10-12) to decompose chronic kidney disease due to diabetes mellitus type 2 attributable to diet high in sugar-sweetened beverages prevalence (death or DALYs) by population age structure, population growth, and epidemiologic changes. The number of prevalence (death or DALYs) at each location was obtained from the following formula:

prevalence (death or DALYs) _ay, py, ey_ =
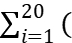
a _i, y_ * p _y_ * e _i, y_)

Where prevalence (death or DALYs) _ay, py, ey_ represented prevalence (death or DALYs) based on the factors of age structure, population, and prevalence (death or DALYs) rate for specific year y; a _i_ _y_ represents the proportion of population for the age category i of the 20 age categories in given year y; p _y_ represents the total population in given year y; and e _i, y_ represents prevalence (death or DALYs) rate given age category i in year y. The contribution of each factor to the change in prevalence (death or DALYs) from 1990 to 2021 was defined by the effect of one factor changing while the other factors were held constant. For example, the effect of age structure was calculated as:

[(DALY _a2021, p1990, e1990_ + DALY _a2021, p2021, e2021_)/3+ (DALY _a2021, p1990, e2021_ + DALY _a2021, p2021, e1990_)/6] - [(DALY _a1990, p2021, e2021_ + DALY _a1990, p1990, e1990_)/3+ (DALY _a1990, p2021b, e1990_ + DALY _a1990, p1990, e2021_)/6]

**8. Data extraction**

We selected “Risk factor” from “GBD Estimate” query box, “Death, and DALYs” from “Measure” query box, and “Number and Rate” from “Metric” query box, “60-64 years, 65-69 years, 70-74 years, 75-79 years, 80-84years, 85-89 years, 90-94 years, 95+ years” from “Age” query box, “Both, Male, and Female” from “Sex” query box, “chronic kidney disease due to diabetes mellitus type 2” from “Cause” query box, “diet high in sugar-sweetened beverages” from “Risk” query box.

**References:**

1. Global incidence, prevalence, years lived with disability (YLDs), disability-adjusted life-years (DALYs), and healthy life expectancy (HALE) for 371 diseases and injuries in 204 countries and territories and 811 subnational locations, 1990-2021: a systematic analysis for the Global Burden of Disease Study 2021. LANCET. [Journal Article]. 2024 2024/5/18;403(10440):2133-61.

2. Global burden of 288 causes of death and life expectancy decomposition in 204 countries and territories and 811 subnational locations, 1990-2021: a systematic analysis for the Global Burden of Disease Study 2021. LANCET. [Journal Article]. 2024 2024/5/18;403(10440):2100-32.

3. Kim HJ, Fay MP, Feuer EJ, Midthune DN. Permutation tests for joinpoint regression with applications to cancer rates. STAT MED. [Journal Article]. 2000 2000/2/15;19(3):335-51.

4. Kim S, Lee S, Choi JI, Cho H. Binary genetic algorithm for optimal joinpoint detection: Application to cancer trend analysis. STAT MED. [Journal Article; Research Support, Non-U.S. Gov't]. 2021 2021/2/10;40(3):799-822.

5. Yang JJ, Trucco EM, Buu A. A hybrid method of the sequential Monte Carlo and the Edgeworth expansion for computation of very small p-values in permutation tests. STAT METHODS MED RES. [Journal Article; Research Support, N.I.H., Extramural]. 2019 2019/10/1;28(10-11):2937-51.

6. Adolescent transport and unintentional injuries: a systematic analysis using the Global Burden of Disease Study 2019. LANCET PUBLIC HEALTH. [Journal Article; Research Support, Non-U.S. Gov't]. 2022 2022/8/1;7(8):e657-69.

7. Fay MP, Feuer EJ. Confidence intervals for directly standardized rates: a method based on the gamma distribution. STAT MED. [Journal Article]. 1997 1997/4/15;16(7):791-801.
